# Stereotype Threat at Work: A Meta-Analysis

**DOI:** 10.1177/01461672241297884

**Published:** 2024-12-13

**Authors:** Courtney von Hippel, Clara Kühner, Sarah P. Coundouris, Amy Lim, Julie D. Henry, Hannes Zacher

**Affiliations:** 1 The University of Queensland, St Lucia, Queensland, Australia; 2 Leipzig University, Germany; 3 Murdoch University, Singapore

**Keywords:** stereotype threat, stigma consciousness, meta-stereotypes, meta-analysis, workplace

## Abstract

Stereotype threat refers to the concern of being judged based on stereotypes about one’s social group. This preregistered meta-analysis examines the correlates of stereotype threat in the workplace (*k* = 61 independent samples, *N =* 40,134). Results showed that stereotype threat was positively related to exhaustion, identity separation, negative affect, turnover intentions, and behavioral coping, and negatively related to career aspirations, job satisfaction, organizational commitment, job engagement, job performance, positive affect, self-efficacy, and work authenticity. In addition, moderator analyses for constructs represented in at least *k* = 10 samples in the focal analyses showed that relations did not differ for measures of stereotype threat and stigma consciousness. However, the negative relationships between stereotype threat and career aspirations, job satisfaction, and job engagement were stronger for older employees compared with female employees as the stereotyped group. Overall, the findings suggest that stereotype threat constitutes an important stressor in the workplace.

Stereotype threat, or the concern of confirming negative stereotypes about one’s social group (Steele, 1997), has been extensively studied in controlled laboratory settings. These studies have demonstrated the acute, detrimental effects of stereotype threat on performance outcomes, particularly in educational and testing environments (e.g., Lamont et al., 2015; Nguyen & Ryan, 2008). Despite the large volume of research showing the negative effects of stereotype threat on performance in experimental settings, two key criticisms have emerged. First, recent meta-analyses have reported varying effect sizes, raising questions about the robustness and consistency of stereotype threat effects (e.g., Picho-Kiroga et al., 2021; Warne, 2022). Second, critics in work and organizational psychology have argued that the performance effects that emerge under laboratory conditions are unlikely to be found in “real world” organizational settings in which the stakes are high (e.g., personnel selection) and/or people are familiar with the domain (i.e., on the job; Shewach et al., 2019).

This skepticism about the consequences of stereotype threat challenges the relevance of this construct beyond the laboratory, particularly in workplace settings where chronic experiences of stereotype threat could have significant implications for outcomes such as job satisfaction, career aspirations, and overall well-being. In contrast to the claims of critics, however, Smerdon et al. (2020) demonstrate how female chess players in high-stakes tournaments underperform due to stereotype threat, illustrating how societal stereotypes can influence performance outcomes even when the consequences are important to individuals in the “real world.” Further evidence that stereotype threat is a real phenomenon can be found in a recent meta-analysis examining the effectiveness of interventions designed to mitigate stereotype threat across various contexts (Liu et al., 2021). Drawing from a wide range of studies, their meta-analysis evaluates the efficacy of interventions, such as educational workshops, stereotype awareness training, and affirmation interventions, in reducing the impact of stereotype threat on performance outcomes. The findings provide support for the effectiveness of these interventions in alleviating stereotype threat effects, suggesting that stereotype threat is indeed a phenomenon that exists outside the laboratory.

In response to the criticisms raised in previous research about the relevance of stereotype threat in the workplace and to gain an understanding of the consistency of stereotype threat effects at work, our meta-analysis makes several key contributions to the literature. First, it provides a comprehensive synthesis of research on stereotype threat in the workplace, addressing criticisms regarding the real-world applicability of stereotype threat effects. Unlike prior reviews that have exclusively focused on performance deficits in laboratory settings (e.g., Picho-Kiroga et al., 2021; Warne, 2022), this meta-analysis examines how stereotype threat, along with the related constructs of stigma consciousness and meta-stereotyping, relates to a wide range of work outcomes. By doing so, our study takes the important next step in examining the relevance of this construct beyond the laboratory and into organizational settings. Second, the findings provide important practical guidance for scholars aiming to conduct studies on stereotype threat in employment settings (e.g., which work outcomes to focus on), as well as initial ideas for organizational practitioners interested in mitigating the detrimental effects of this pervasive phenomenon (e.g., through interventions). Finally, by incorporating the related constructs of stigma consciousness and meta-stereotyping, this meta-analysis helps clarify the distinctiveness and potential overlap between these concepts and their potential differential association with work-related outcomes.

## Stereotype Threat and Its Potential Effects in the Workplace

Stereotype threat was initially proposed to understand the underperformance of stereotyped groups in certain tasks (Steele, 1997). Most stereotype threat research has been conducted in laboratory settings with tightly controlled manipulations that focus on performance deficits (Spencer et al., 2016). Studies such as Steele and Aronson’s (1995) seminal work on African American students’ academic underperformance have contributed to our understanding of how situational cues, such as being asked to indicate their race/ethnicity, or being told the test is diagnostic of intellectual ability, can disrupt individuals’ performance in domains where negative stereotypes about their group are salient. This body of research, spanning nearly three decades, has demonstrated the detrimental impact of stereotype threat on various cognitive tasks, including standardized tests and academic assessments (Spencer et al., 2016).

Subsequent research has expanded our understanding of the mechanisms and implications of stereotype threat. Key contributions from several scholars have enriched the theoretical framework, providing insights into how stereotype threat operates in different contexts and populations. For example, Cohen and Garcia (2008) have emphasized the significance of identity contingencies—the situational cues that signal whether an individual’s social identity will be respected or stigmatized in a given environment. Their research suggests that when individuals perceive their environment as identity-threatening, it can trigger stereotype threat, leading to decreased performance, disengagement, and even long-term withdrawal from the threatening domain. This perspective highlights the importance of creating identity-affirming environments as a means of mitigating the detrimental effects of stereotype threat.

Schmader et al. (2008) developed an integrated process model that explains how stereotype threat disrupts performance through a combination of cognitive, affective, and physiological processes. According to Schmader et al.’s model, the experience of stereotype threat taxes working memory, heightens emotional regulation efforts, and triggers stress responses, all of which collectively impair an individual’s ability to perform effectively. Although not directly stated in their model, these three processes should also play an important role beyond performance, by causing the stereotyped domain to be experienced as less rewarding over time. This model can therefore be extended to predict that stereotype threat should lead to more negative work attitudes, particularly in high-stakes environments such as the workplace, where stressors are common.

Research by Ryan and colleagues has extended the understanding of stereotype threat into organizational contexts, focusing on how it impacts diversity and inclusion within the workplace. Ryan and Wessel (2012) have explored how stereotype threat influences critical organizational processes such as hiring, promotion, and employee retention. Their research underscores the role of organizational practices and leadership in either exacerbating or alleviating stereotype threat, particularly through the implementation of diversity initiatives and inclusive policies that foster a sense of fairness and belonging among employees.

Aligned with these findings, in addition to proposing that stereotype threat would result in acute disruptions to performance, Steele (1997) initially theorized that stereotype threat may have longer-term consequences for domain identification (e.g., math, work). Steele (1997) proposed that individuals subjected to chronic experiences of stereotype threat would disidentify or disengage from the domain in which stereotype threat is present. Disidentification refers to the process through which individuals detach their self-esteem from the outcomes in the threatened domain, thereby protecting their self-concept from the negative implications of stereotype threat (Steele, 2011). Disengagement is typically a result of disidentification and involves withdrawing effort or investment from the threatened domain, in part because the domain is no longer self-defining and in part to avoid confirming the negative stereotype about one’s group (Crocker, 2002). These responses to stereotype threat protect self-esteem but may ultimately contribute to reduced motivation, achievement, and well-being in the targeted domain over time (Crocker, 2002; Steele, 1997).

Consistent with Steele’s theorizing, a substantial body of research now demonstrates that chronic feelings of stereotype threat are associated with more negative job attitudes and reduced job engagement (e.g., Casad & Bryant, 2016; Kalokerinos et al., 2014; L. Roberson & Kulik, 2007; Swab et al., 2022). For example, studies by von Hippel et al. (2013, 2019) have found that stereotype threat is associated with lower job satisfaction and increased intentions to quit among women in male-dominated fields and among older employees. Furthermore, longitudinal research by Kulik et al. (2016) has shown how stereotype threat is associated with diminished workplace engagement. Other research demonstrates that female engineers experience stereotype threat from interactions with their male colleagues, which in turn led to increased exhaustion and burnout (Hall et al., 2015; see also von Hippel et al., 2023 for similar research among older employees interacting with younger colleagues). These findings underscore the pervasive nature of chronic stereotype threat across different domains and highlight its relevance for understanding workplace dynamics among different groups of stereotyped employees (e.g., female and older employees).

## Stereotype Threat, Stigma Consciousness, and Meta-Stereotyping

Although research on stereotype threat is typically conducted independently from work on related constructs, there are two additional constructs that are conceptually related: stigma consciousness and meta-stereotyping. All three constructs focus on stereotyping, but they each address different aspects of how individuals experience and respond to stereotypes. Stereotype threat is the fear that one’s actions will confirm negative stereotypes about one’s social group, especially in situations where these stereotypes are salient (Steele, 1997).

Stigma generally refers to the process by which individuals or groups are socially discredited based on certain attributes, such as race, gender, age, or mental health (Major & O’Brien, 2005). This process often leads to the development of negative stereotypes, prejudice, and discrimination, which can result in significant psychological distress, reduced self-esteem, and social isolation for those who are stigmatized (Major & O’Brien, 2005). Stigma consciousness differs from stereotype threat in that it reflects a chronic awareness of, and sensitivity to, the stereotypes associated with one’s social group. Individuals high in stigma consciousness are more likely to perceive prejudice and discrimination in their environment, which can lead to anticipatory stress and defensive behaviors (Pinel, 1999). Unlike stereotype threat, which is typically situationally induced, stigma consciousness is conceptualized as an enduring trait that influences how individuals interpret and react to their surroundings on a day-to-day basis.

In the laboratory, the situational vs. dispositional aspects of stereotype threat and stigma consciousness may lead to divergent approaches. In contrast, in the workplace, where regular acute experiences of stereotype threat can lead to chronic levels of stereotype threat (e.g., Coulon et al., 2024; Kalokerinos et al., 2014), the distinction between the two constructs might become blurred. Moreover, the workplace presents unique conditions that distinguish it from other settings where stereotype threat has been studied. In the workplace, the implications of stereotype threat are not confined to short-term performance but can have long-lasting effects on an individual’s career experiences, job satisfaction, and overall well-being (Kalokerinos et al., 2014).

Meta-stereotyping refers to the awareness and consideration of how one’s own group is perceived by others, which may lead individuals to alter their actions based on how they believe others are judging them (Vorauer et al., 1998). For example, older employees may think about how older workers typically are perceived by others (e.g., as less adaptable) and, accordingly, may act less confidently in situations involving organizational changes (Finkelstein et al., 2020). Although the focus of meta-stereotyping and stereotype threat differ, awareness and reaction to the stereotypes of others is a key component of both theories and a domain in which both theories make similar predictions. Thus, while these constructs are theoretically distinct, they may operate via similar psychological mechanisms, such as increased stress, heightened vigilance, and shifts in self-concept (Voyles et al., 2014). These mechanisms may manifest in various ways in the workplace, potentially influencing outcomes such as job satisfaction, commitment, performance, and turnover intentions. In summary, despite their distinct focuses, stereotype threat, stigma consciousness, and meta-stereotyping overlap in their impact on individuals’ psychological experiences and behaviors related to stereotypes, showing how pervasive and multifaceted the influence of stereotypes can be. Given this theoretical overlap, even though the literatures on these constructs tend to be studied in isolation, we included stigma consciousness and meta-stereotyping in our meta-analysis.

## Stigmatized Identities

Stigmas in the workplace can take various forms, such as gender, age, or family obligations. Gender stigma is primarily focused on women as the stigmatized group, given the substantial body of literature that explores stereotype threat experienced by women in various professional settings (e.g., von Hippel, 2012). Age-related stigma is another key focus, examining how negative stereotypes about older or younger employees impact their workplace experiences (Posthuma et al., 2009). Prior literature has assessed the extent to which employees felt threatened by stereotypes about their age group, which could influence factors such as job satisfaction and intentions to quit. Age-based stereotype threat may be more salient in contexts where youth is highly valued, potentially leading older employees to feel vulnerable to stereotypes about declining abilities (von Hippel et al., 2019). Finally, family obligations stigma refers to the negative stereotypes associated with individuals who have significant caregiving responsibilities, such as parents or those caring for older relatives (Calvano, 2013). This form of stigma often manifests in perceptions that these individuals are less committed to their jobs or less capable of fulfilling workplace duties (Cuddy et al., 2004), which can lead to experiences of stereotype threat.

Different stigmatized identities—such as gender and age—might relate differentially to various workplace outcomes. For example, gender-based stereotype threat could influence women’s participation and advancement in male-dominated fields such as STEM (science, technology, engineering, and mathematics), whereas age-based stereotype threat may affect older workers’ engagement, productivity, and career longevity. Given these potential variations, we investigate whether type of stigmatized identity moderates the relationships between stereotype threat and related constructs and workplace outcomes. This approach allows us to explore whether certain identities are more vulnerable to the effects of stereotype threat.

## The Present Study

The phenomenon of stereotype threat has been extensively studied in controlled laboratory settings. More recently, research has demonstrated the relevance of stereotype threat in real-world settings, such as the workplace. Workplace environments present unique challenges that differ significantly from the controlled conditions of the laboratory. Employees face ongoing pressures, complex interpersonal dynamics, and the potential for long-term consequences that extend beyond immediate performance. As such, understanding how stereotype threat relates to workplace outcomes is essential for determining its broader relevance.

While a single study may not be sufficient to establish the real-world relevance of stereotype threat, a body of literature can collectively address this question. The primary goal of this preregistered meta-analysis is to consolidate and systematically integrate the existing research on stereotype threat in the workplace. By synthesizing findings across multiple studies, this meta-analysis aims to provide a more comprehensive and reliable estimate of the relationships between stereotype threat, stigma consciousness, and meta-stereotyping, respectively, and various work-related outcomes, such as job satisfaction, turnover intentions, and career aspirations. This approach allows us to quantify the magnitude and direction of stereotype threat’s relations with workplace outcomes and to identify whether consistent patterns emerge across different contexts (e.g., age- vs. gender-based stereotype threat). In doing so, this meta-analysis contributes to a deeper understanding of the potential role of stereotype threat in workplace dynamics and outcomes, addressing the critical issue of its relevance beyond the laboratory.

As research on stereotype threat has evolved, the relationship between stereotype threat and a large variety of workplace outcomes has been examined (e.g., job satisfaction, organizational commitment, turnover intentions, occupational well-being). Consistent with methodological recommendations (Rudolph et al., 2020), this meta-analysis includes all possible workplace outcomes that have been investigated in relation to stereotype threat, stigma consciousness, and/or meta-stereotypes in three or more independent samples. Workplace outcomes are assessed not as a unitary construct (i.e., aggregated), but are subdivided into meaningful synthetic constructs (see Table 1 for an overview) to gain a more nuanced understanding of how stereotype threat relates to workplace outcomes.

**Table 1.** Synthetic Constructs Considered as Potential Consequences of Stereotype Threat.

Synthetic construct	Example operationalization
Behavioral coping	
Dysfunctional behavioral coping	Isolation, avoidance, alcohol use
Functional behavioral coping	Feedback seeking, redefine criteria for success, choose battles
Career-related	
Career aspirations	Career aspirations, occupational future time perspective, intentions to advance
Career opportunities	Promotional opportunities, perceived negative career consequences (reverse coded)
Job attitudes	
Organizational commitment	Organizational commitment, affective commitment, belonging
Job satisfaction	Job satisfaction, career recommendation, opportunity satisfaction
Performance	
Job engagement	Job engagement, job involvement, thriving at work
Job performance	Job performance, in-role behaviors, workplace cognitive failure (reverse coded)
Organizational citizenship behavior	Organizational citizenship behavior
Well-being	
Exhaustion	Emotional exhaustion, burnout, self-depletion
Identity separation	Identity separation, leader disidentification, occupational identification (reverse coded)
Negative affect	Negative emotions, negative mood
Positive affect	Positive emotions, positive mood
Self-efficacy	Self-efficacy, competence, psychological capital
Work authenticity	Work authenticity
Work withdrawal	
Deviant behavior	Missed work, organizational production deviance, deviant behavior
Turnover intentions	Turnover intentions, intention to quit, intention to retire

We also explore whether the publication year of the studies moderates the relationship between stereotype threat and workplace outcomes. The rationale for this is threefold. First, awareness of and responses to feeling stigmatized may have evolved over time, particularly as discussions around diversity and inclusion have gained prominence in organizational contexts (Casad & Bryant, 2016). For example, women are increasingly represented in numerous (but not all) areas that were once male-dominated, such as accounting and medicine (Blau et al., 2013), raising the possibility that gender-based stereotype threat might dissipate in some domains over time. Of course, we may also see similar effects with age-based stereotype threat in the future, as the population ages and older workers become more normative (Rothermund et al., 2021). Second, interventions aimed at reducing discrimination in the workplace have become more widespread in recent years (Roberson et al., 2020), potentially altering the strength or nature of the relationship between stereotype threat and related constructs and workplace outcomes. These interventions may alter targets of stereotypes sensitivity to stereotyping. Finally, it is possible that research methods to study workplace phenomena have improved over time (e.g., Cortina et al., 2017), leading to more accurate and robust findings in later studies.

In terms of our preregistered predictions (see https://osf.io/m3y7u/), we generally expected that employees who report more stereotype threat will experience more negative workplace outcomes. Thus, we expected associations between stereotype threat in the workplace and several, commonly investigated work-related variables (Table 1). First, we assumed that stereotype threat is a stressor that would be positively related to more dysfunctional (e.g., alcohol use), and negatively related to functional forms of *behavioral coping* (e.g., feedback seeking). Second, we hypothesized that stereotype threat would be negatively related to favorable *career-related variables*, including career aspiration (e.g., intentions to advance in one’s career) and career opportunities (e.g., promotions). Third, we likewise assumed that stereotype threat is negatively related to two commonly studied, favorable *job attitudes*, organizational commitment and job satisfaction. Fourth, we expected that stereotype threat is negatively related to different aspects of employees’ *work performance*, including job engagement (i.e., being highly involved in one’s work role), job performance (i.e., behavior that contributes to the organization’s goals), and organizational citizenship behaviors (i.e., discretionary behavior that support organizational functioning). Fifth, we expected that stereotype threat is negatively related to various positive *well-being* outcomes, including positive affect, self-efficacy, and work authenticity, and positively related to negative well-being or strain outcomes, including exhaustion, identity separation, and negative affect. Finally, we proposed that stereotype threat would be positively associated with higher *withdrawal from work*, including deviant behavior (e.g., missing work) and turnover intentions (i.e., the intent to quit).

## Method

### Search Strategy

**Figure 1.**
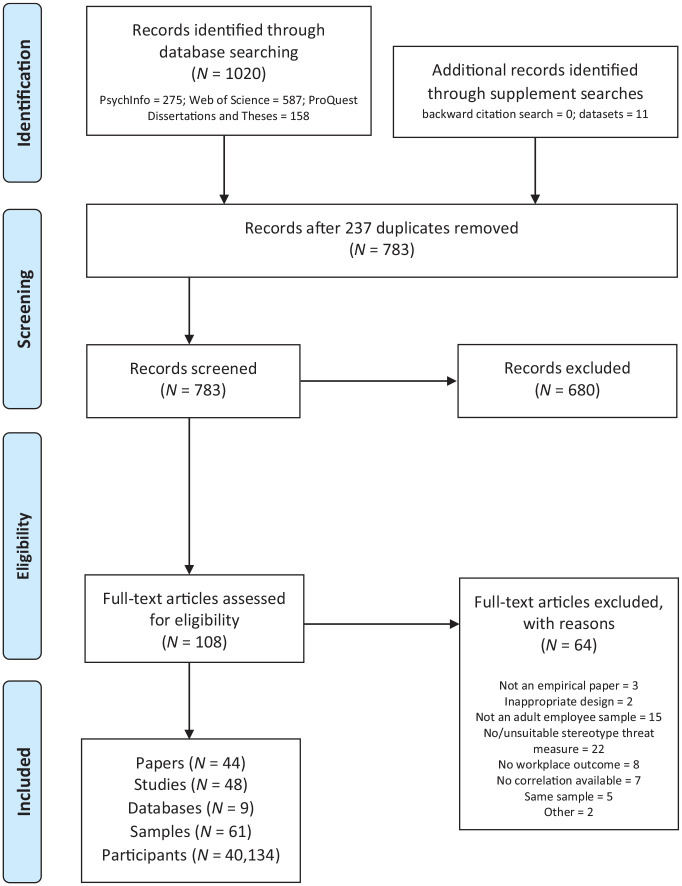
PRISMA Flowchart.

This meta-analysis was preregistered at the Open Science Framework (OSF; see https://osf.io/m3y7u/ for the preregistration and all supplementary materials), and adhered to PRISMA (Preferred Reporting Items for Systematic Reviews and Meta-Analyses) guidelines (Page et al., 2021; see Figure 1 for PRISMA flowchart). A systematic literature search of PsycINFO, Web of Science, as well as ProQuest Dissertations and Theses Global electronic databases was completed in November 2022. The search terms comprised two clusters (stereotype threat and workplace outcomes). Specifically, the key terms searched were: stereotype threat, social identity threat, meta?stereotyp*, stigma consciousness, stereotype lift, or stereotype boost; and worker, workplace, job, employ*, or organi?ation*. This systematic search was supplemented by (a) a backward citation search for additional studies within nine identified relevant reviews (i.e., Casad & Bryant, 2016; Emerson & Murphy, 2014; Hoyt & Murphy, 2016; Kalokerinos et al., 2014; Kray & Shirako, 2012; Swab et al., 2022; von Hippel, 2012; Walton et al., 2015), (b) a call for unpublished data on relevant listservs, and (c) an email request for unpublished data to authors who have published two or more papers in this area.

### Search Eligibility Criteria

All titles and abstracts were initially screened for clear ineligibility (i.e., not on topic, not an empirical paper, a case or qualitative study, not an adult employee sample, and/or an experimental study whereby stereotype threat was manipulated rather than measured). As per our preregistration, a second rater checked agreement on 25% of studies, to ensure high inter-rater reliability (Cohen’s *K* > .90); this requirement was met (*K =* .94).

The entire full-text screening process was completed independently by two of the authors (*K =* .91). In instances of uncertainty, inclusion was discussed with the other authors. Studies were considered eligible if they met four criteria. First, studies had to be quantitative investigations that report original data. Accordingly, qualitative studies, systematic reviews, and book chapters (unless they included quantitative data not reported elsewhere) were excluded. In addition, case studies were considered ineligible. Second, studies had to include samples of employees assessed in relation to their workplace. As such, samples comprising university students (including students for a specific career) were excluded. Third, studies had to measure both stereotype threat or related constructs and at least one potential workplace outcome fitting into our synthetic constructs (see Table 1). Because of our focus on more chronic experiences of stereotype threat, studies that manipulated rather than measured stereotype threat were excluded. Fourth, the statistics had to be published or provided by the author upon request and could be used to calculate a precise relationship effect size for the stereotyped group. Finally, since nondependency of effect sizes is required for meta-analyses (Borenstein et al., 2021), we excluded studies where the same correlations were reported in more than one published study.

### Definition and Operationalization of Variables

#### Stereotype Threat

*Stereotype threat* (also known as social identity threat) is typically manipulated in the lab, often by reminding participants about their membership in a stereotyped group or the underperformance of their group (e.g., Spencer et al., 1999). In the workplace, there can be subtle, yet frequent, reminders that create a more chronic state of stereotype threat for stigmatized groups (Kalokerinos et al., 2014). The focus of this meta-analysis was stereotype threat that has been measured (typically using self-reports provided by employees), not manipulated. Most measures of stereotype threat assess individuals’ concern about confirming negative stereotypes or employees’ beliefs that they are being judged based on negative stereotypes about their group. For example, in their seminal research on stereotype threat, Steele and Aronson (1995) measured stereotype threat by asking participants questions like “Some people think I have less verbal ability because of my race” and “The experimenter expected me to do more poorly because of my race” (see Study 4). These items have been adapted to a workplace to tap more chronic or recurrent feelings of stereotype threat by asking questions such as “Some of my colleagues feel I’m not as committed because of my age (gender)” (e.g., von Hippel et al., 2013).

As noted, given the high degree of conceptual and empirical overlap with the constructs *stigma consciousness* (the degree to which people focus on their stereotyped status) and *meta-stereotypes* (beliefs about how people from other groups stereotype members of your own group), these constructs were also included. The Stigma Consciousness Questionnaire (SCQ; Pinel, 1999) was designed to measure an individual’s chronic awareness of stereotypes about their social group and the potential impact of these stereotypes on their daily lives. This measure has been widely adopted in research exploring the effects of stigma consciousness across various domains. In workplace contexts, the SCQ assesses how individuals perceive and respond to the possibility of being stereotyped by colleagues, supervisors, or clients. Meta-stereotype measures ask participants how they believe their social group is viewed by other groups in terms of certain negative attributes. For example, to assess age-related meta-stereotypes, older workers are asked, “How do you think members of your age group are viewed by members of other age groups? Technophobic, slow, out of touch, narrow minded” (Finkelstein et al., 2020). Consistent with our preregistered plans, the specific construct assessed was coded to allow for a comparison of relationships.

In our analysis, we adhered to the original authors’ interpretations of measurement scales, even when the same scale was used to assess different constructs. This decision impacted two studies. Specifically, Allen et al. (2021) utilized a scale originally designed to measure stereotype threat but referred to it as capturing meta-stereotyping. In this instance, we classified it based on the original authors’ intended use (i.e., we classified the scale as stereotype threat). Steffens et al. (2019) also measured stereotype threat using Steele and Aronson’s measure, but in their paper the authors refer to it as capturing stigma consciousness. Here again we classified the scale as stereotype threat. This method ensured that our synthesis of the literature was grounded in the original conceptual frameworks, enhancing accuracy and consistency.

Finally, while an original additional aim of this review was to address the positive side of stereotype threat, that is *stereotype lift* (or the boost in performance experienced by members of the nonstigmatized group) and *stereotype boost* (the boost in performance caused by positive stereotypes about your group), due to the few studies (i.e., *k* = 2) identified that considered this topic (i.e., Finkelstein et al., 2020; Ryan et al., 2015), these data did not contribute to the meta-analysis.

#### Workplace Outcomes

Scholars have examined relationships between stereotype threat and a large variety of workplace outcomes, including job attitudes, occupational well-being, and work behaviors. This meta-analysis included all possible workplace outcomes that had been measured in at least three or more studies on stereotype threat. Workplace outcomes were divided into synthetic construct groupings (see Table 1). This categorization occurred once the full list of outcomes was identified. This was achieved by having two authors first independently develop specific outcome subcategories that would be most meaningful, before discussing these proposed subcategories together. As may be seen in Table 1, 17 synthetic constructs were created, that are captured by six broader categories (i.e., behavioral coping, career-related outcomes, job attitudes, performance, well-being, and work withdrawal).^1^

### Quality Assessment

In line with PRISMA recommendations (Page et al., 2021), the appraisal tool for cross-sectional studies (AXIS; Downes et al., 2016) was used to assess the quality of each individual study. This tool typically involves dichotomously assessing each study on 20 criteria (yes, classified as one; or no/unclear, classified as zero). However, in the present meta-analysis, three questions regarding the categorization and description of nonresponders were removed. This was because the use of online recruitment for most participants in the contributing studies made these categorization decisions difficult to apply. As the AXIS does not provide specific interpretation guidelines, there is a degree of subjectivity involved, but this has been argued to be a potential strength as it allows for greater flexibility (Downes et al., 2016). In the present meta-analysis, study quality was examined within the subcategories of reporting (seven questions), design (seven questions) and potential biases (three questions), with studies characterized as low (0–3 times “yes” for reporting and design, 0–1 times “yes” for biases), medium (4–5 times “yes” for reporting and design, 2 times “yes” for biases), or high quality (6–7 times “yes” for reporting and design, 3 times “yes” for biases; question 19 reverse scored) for each category. When a paper reported multiple studies (e.g., von Hippel et al., 2011), each relevant study was assessed separately. The quality assessment was introduced in moderator analyses as detailed below.

### Data Extraction

The following data were extracted:

(1) *Participant characteristics*. Mean age, percentage of females in the sample, occupation, industry type, industry mean age, industry gender domination, and stigmatized group (e.g., females, older workers).
(2) *Study characteristics*. Sample size, country, year of publication, publication status, and research design (e.g., cross-sectional, longitudinal, diary).
(3) *Stereotype threat (and related constructs) measure characteristics*. Paper and meta-classification of construct, measure used, and reliability estimate.
(4) *Workplace outcome measure characteristics*. Paper classification, meta-synthetic construct classification (see Table 1), measure used, and reliability estimate.
(5) Correlation coefficients and sample sizes.

For longitudinal study designs (e.g., Lavaysse & Probst, 2021) and experience sampling/diary study designs (e.g., Finkelstein et al., 2020; von Hippel et al., 2019), we extracted relationships based on time one data only. If correlations were unavailable, authors were contacted. If authors did not respond, we excluded the respective study. We standardized the direction of correlation coefficients between studies to produce consistent meanings of effect sizes. When studies reported results from multiple independent samples, we included each sample separately in the meta-analysis (e.g., von Hippel et al., 2013, Study 1, reported results on a sample of media company employees and results on a sample of law enforcement employees).^2^

To determine inter-rater reliability of coding, a second coder recoded a random sample of 10 of the 48 included studies (20%). Inter-rater agreement was very high for zero-order correlations (93.45 %). The few disagreements were due to misunderstandings of the coding direction (e.g., omitting to reverse the sign of the relationship between stereotype threat and self-efficacy when a study measured inefficacy). All coder disagreements were reconciled via consensus discussions until agreement was reached.

### Statistical Analyses

We followed random-effects procedures described by Schmidt and Hunter (2015) to statistically integrate the data. We corrected effect sizes for sampling and measurement error of both stereotype threat and workplace outcomes. Sampling error was corrected by estimating sample-size weighted mean effect sizes *r*. We used reliability estimates (i.e., Cronbach’s alpha) for stereotype threat and the workplace outcomes, respectively, to correct for measurement error. In a few studies, reliability information was not made available for a specific measure. Since we applied artifact distribution meta-analyses, no imputation of missing reliability estimates was required (Wiernik & Dahlke, 2020).^3^ In cases of dependency created from a study reporting multiple effects sizes contributing to the same overall effect size estimate (e.g., a study assessing stereotype threat using two different measures; Borenstein et al., 2021), we calculated composite effect sizes by using the composite formula 10.6 by Schmidt and Hunter (2015, p. 442) and computed composite reliabilities applying the Mosier formula (Mosier, 1943). The interpretation of effect sizes follows recommendations by Bosco et al. (2015), who classify uncorrected correlations involving behavioral variables between | *r* | = 0.10 and 0.25 as medium-sized. Statistical significance at *p* < .05 (two-tailed) is established when the 95% confidence interval (CI) around the effect size does not include zero. All analyses were conducted using the packages *psychmeta* (Dahlke & Wiernik, 2019) and *metafor* (Viechtbauer, 2010) for the R statistical computing environment. Several heterogeneity metrics were calculated including the *Q* statistic, the percentage of the true effect size heterogeneity within the total variance of effect sizes (*I*^2^), and the percentage of variance attributable to sampling error and statistical artifacts (% Var).

#### Sensitivity Analyses

Two sensitivity techniques were used to identify any effect sizes that may be exerting disproportionate influence on the overall findings. Cook’s distances (Cook & Weisberg, 1982) measure the effect of deleting one study for a given effect size and values should not be larger than 0.45 (Viechtbauer & Cheung, 2010). We complemented this analysis by examining how average effect sizes would change if one study out of the whole set of studies for each relationship were excluded from the analysis in so-called “leave-one-out” analyses. In addition, we calculated standardized residuals where scores ± 2.24 standard deviations were regarded as extreme (Aguinis et al., 2013; Martin & Roberts, 2010). Once a potentially influential case was identified, a new summary effect excluding this case was calculated and compared with the original to assess any potential distortion to the pooled effect (Viechtbauer & Cheung, 2010). Furthermore, we considered cumulative meta-analyses to address whether studies with lower precision (i.e., smaller N) cause “drift” in our meta-analytic estimates (McDaniel, 2009). In line with common practice (e.g., Rudolph et al., 2017), we conducted sensitivity analyses for the “highest K” constructs represented in at least *k* = 10 studies in the focal analyses (i.e., turnover intentions, job satisfaction, identity separation, job engagement, organizational commitment, functional behavioral coping, job performance, and career aspirations).

#### Moderator Analyses

We considered two categorical moderators of the relationships between stereotype threat and workplace outcomes (i.e., classification of stereotype threat: stereotype threat vs. stigma consciousness; stigmatized group: females vs. older workers). We conducted subgroup analyses for categorical moderators that contained at least *k* = 3 studies per category and checked CIs of effect sizes in the subgroups for overlap. In cases where 95% CIs do not overlap, a moderation effect is likely present. Comparison of the CIs for overlap is the recommended approach for testing categorical moderators and serves as a stringent and reliable test (Hwang & Schmidt, 2011; Morris, 2023; Rudolph et al., 2020; Schmidt, 2017; Schmidt & Hunter, 2015). There were not enough studies considering meta-stereotyping in either of the relationships, so we were only able to compare stereotype threat and stigma consciousness. In addition, we specified meta-regression models to identify the moderating effect of study quality (Morris, 2023).

#### Tests for Publication Bias

Presence of publication bias was assessed through Egger’s test (Egger et al., 1997) and the trim-and-fill procedure (Duval & Tweedie, 2000). To supplement these tests, we assessed the potential moderating effects of two publication characteristics: year of publication and publication status (published vs. unpublished). We introduced publication status as a categorical moderator and specified meta-regression models to identify the moderating effect of publication year. In line with common practice (e.g., Rudolph et al., 2017), we conducted these tests for “highest K” constructs represented in at least *k* = 10 studies in the focal analyses (i.e., turnover intentions, job satisfaction, identity separation, job engagement, organizational commitment, functional behavioral coping, job performance, and career aspirations).

## Results

### Sample Description

As detailed in Figure 1, our final sample consisted of 44 manuscripts representing 48 studies. Of the included 44 manuscripts, six were dissertation theses. In addition, we included nine unpublished data sets. Overall, our sample consisted of 57 studies, comprising 61 independent samples and *N* = 40,134 participants. Table 2 provides a list of the studies and data sets included in the meta-analysis. Included studies were from 13 different countries, with the majority (*N* = 21) being conducted in the United States. The mean age of participants was *M* = 42.59 years (*SD* = 11.44) and on average *M* = 67.63% (*SD* = 31.48) of the samples were female. Most studies (*N* = 41) were cross-sectional, whereas only *N* = 3 studies used a diary approach and *N* = 4 studies used a longitudinal design. Participants were stigmatized due to gender, age, ethnicity, profession, or family obligations. In moderator analyses, however, we were only able to consider females and older workers as stigmatized groups since the number of studies was below three for the other groups.

**Table 2.** Studies Included in Meta-Analysis With Stereotype Threat Classification, Stigmatized Group and Synthetic Constructs.

Study	Stereotype threat classification	Stigmatized group	Synthetic constructs
Allen et al. (2021)	Stereotype threat	Older age	Job engagement
Amin et al. (2023)	Stereotype threat	Profession	Functional behavioral coping Organizational commitment
Bal et al. (2015)	Meta-stereotype	Older age	Career aspirations Turnover intentions
Bazemore et al. (2010)	Stigma consciousness	Other	Organizational commitment Job performance
Bedyńska & Żołnierczyk-Zreda (2015)	Stereotype threat	Female gender	Job engagement Exhaustion Negative affect Positive affect
Charles (2009)	Stereotype threat	Ethnicity	Functional behavioral coping Self-efficacy
Chung et al. (2010)	Stereotype threat	Ethnicity	Job performance
Collins (2007)	Stereotype threat	Other	Functional behavioral coping Job performance Self-efficacy
Cortland & Kinias (2019)	Stereotype threat	Female gender	Job satisfaction
Cruz & Nagy (2022)	Stigma consciousness	Female gender	Dysfunctional behavioral coping Functional behavioral coping Job satisfaction Negative affect Turnover intentions
Cruz [Unpublished dataset Nr. 1]	Stereotype threat Stigma consciousness	Female gender	Organizational commitment Job satisfaction Job performance Turnover intentions
Cruz [Unpublished dataset Nr. 2]	Stereotype threat Stigma consciousness	Female gender	Functional behavioral coping Job satisfaction Identity separation Negative affect Turnover intentions
Cyr et al. (2021)	Stereotype threat	Female gender	Organizational commitment Identity separation Self-efficacy
da Silva Oliveira & Cabral-Cardoso (2017)	Stereotype threat	Older age	Organizational commitment
da Silva Oliveira (2021)	Stereotype threat	Younger age, older age	Functional behavioral coping Job engagement Turnover intentions
Deery et al. (2013)	Stigma consciousness	Profession	Career opportunities Job satisfaction Job performance Turnover intentions
Finkelstein et al. (2020)	Stereotype threat Meta-stereotype	Older age	Dysfunctional behavioral coping Functional behavioral coping Job engagement
Gordon [Unpublished dataset]	Stereotype Threat	Female gender	Job satisfaction Job engagement Positive affect Turnover intentions
Guidetti et al. (2021)	Stereotype Threat	Profession	Exhaustion
Hall et al. (2019)	Stereotype threat Stigma consciousness	Female gender	Organizational commitment Exhaustion Self-efficacy
Jarunratanakul & Jinchang (2018)	Stereotype threat	Female gender	Career aspirations Identity separation
Kalokerinos et al. (2017)	Stereotype threat	Male gender	Organizational commitment Job satisfaction Turnover intentions
Kulik et al. (2016)	Stereotype threat	Older age	Job engagement
Lavaysse (2021)	Stereotype threat	Family	Job performance Organizational citizenship behavior
Lavaysse & Probst (2021)	Stereotype threat	Family	Dysfunctional behavioral coping Functional behavioral coping Job performance
Locke (2016)	Stereotype threat	Female gender	Career aspirations Self-efficacy Turnover intentions
Manzi et al. (2019)	Stereotype threat	Older age	Career aspirations Job engagement Identity separation
Manzi, Coen et al. (2021)	Stereotype threat	Older age	Organizational commitment Job engagement Work authenticity
Manzi, Sorgente et al. (2021)	Stereotype threat	Older age, female gender	Organizational commitment Job performance Work authenticity
Manzi [Unpublished dataset]	Stereotype threat	Older age, female gender	Career aspirations Job engagement Job performance Identity separation
McIntosh et al. (2023)	Stigma consciousness	Other	Job engagement
Membere (2018)	Stereotype threat	Other	Career aspirations Job performance
Miller (2019)	Stereotype threat	Family	Functional behavioral coping Deviant behavior Turnover intentions
Newland [Unpublished dataset]	Stereotype threat	Female gender	Job satisfaction Organizational citizenship behavior Exhaustion Positive affect Turnover intentions
Pickern (2017)	Stigma consciousness	Other	Job satisfaction
Pinel & Paulin (2005)	Stigma consciousness	Female gender	Self-efficacy Turnover intentions
Reilly et al. (2019)	Stigma consciousness	Female gender	Job engagement Turnover intentions
Roberson et al. (2003)	Stereotype threat	Ethnicity	Dysfunctional behavioral coping Functional behavioral coping
Ryan et al. (2015)	Stigma consciousness	Younger age	Dysfunctional behavioral coping Functional behavioral coping Job satisfaction Negative affect
Schmader & Bergsieker [Unpublished dataset]	Stereotype threat	Female gender	Organizational commitment Exhaustion Identity separation Work authenticity Turnover intentions
Shantz & Booth (2014)	Stigma consciousness	Profession	Deviant behavior
Tooley [Unpublished dataset]	Stereotype threat Stigma consciousness	Older age	Organizational commitment Job satisfaction Organizational citizenship behavior Exhaustion Turnover intentions
Velez et al. (2013)	Stigma consciousness	Other	Dysfunctional behavioral coping Functional behavioral coping Job satisfaction
von Hippel et al. (2011)	Stereotype threat	Female gender	Career aspirations Job satisfaction Identity separations Turnover intentions
von Hippel et al. (2013)	Stereotype threat	Older age	Organizational commitment Job satisfaction Positive affect Turnover intentions
von Hippel et al. (2015)	Stereotype threat	Female gender	Job satisfaction Identity separation Negative affect
von Hippel et al. (2017)	Stereotype threat	Family, female gender	Career opportunities Functional behavioral coping
von Hippel et al. (2019)	Stereotype threat	Older age	Dysfunctional behavioral coping Functional behavioral coping Organizational commitment Job satisfaction Job engagement Positive affect Turnover intentions
von Hippel [Unpublished dataset]	Stereotype threat	Older age, female gender	Organizational commitment Job satisfaction Job performance Turnover intentions
Wells et al. (2020)	Stigma consciousness	Female gender	Job satisfaction
Wildes (2005)	Stigma consciousness	Profession	Job satisfaction Turnover intentions
Wong [Unpublished dataset]	Stereotype threat Stigma consciousness	Female gender	Organizational commitment Job satisfaction Job engagement Deviant behavior Turnover intentions
Zhao & Zhan (2022)	Stigma consciousness	Profession	Exhaustion Deviant behavior

### Relationships Between Stereotype Threat and Workplace Outcomes

Meta-analytic results for relationships between stereotype threat and workplace outcomes are summarized in Table 3.

**Table 3.** Meta-Analytic Estimates of the Relations Between Stereotype Threat and Workplace Outcomes.

Variable	*k*	*N*	$\bar{r}$	$SD_{r}$	$SD_{res}$	$\bar{\rho }$	$SD_{r_{c}}$	$SD_{\rho }$	95% CI	80% CR	% Var
Behavioral coping											
Dysfunctional behavioral coping	7	1,835	.19	.19	.18	.24	.24	.23	(0.02, 0.46)	(−0.09, 0.57)	10.31
Functional behavioral coping	15	3,785	.12	.21	.20	.15	.25	.24	(0.01, 0.28)	(−0.18, 0.47)	9.34
Career-related											
Career aspirations	10	11,568	−.19	.09	.09	−.25	.12	.11	(−0.34, −0.17)	(−0.41, −0.09)	10.83
Career opportunities	3	749	−.33	.14	.12	−.44	.19	.17	(−0.91, 0.03)	(−0.75, −0.12)	23.81
Job attitudes											
Organizational commitment	17	17,608	−.25	.09	.09	−.30	.11	.11	(−0.36, −0.24)	(−0.45, −0.16)	12.02
Job satisfaction	22	6,374	−.28	.14	.13	−.34	.17	.15	(−0.42, −0.27)	(−0.54, −0.14)	16.83
Job performance											
Job engagement	15	18,498	−.23	.07	.07	−.29	.10	.09	(−0.35, −0.24)	(−0.41, −0.17)	18.12
Job performance	12	19,972	−.28	.08	.07	−.35	.10	.09	(−0.41, −0.29)	(−0.48, −0.23)	12.94
Organizational citizenship behavior	3	900	.13	.14	.13	.16	.18	.16	(−0.28, 0.61)	(−0.14, 0.47)	17.06
Well-being											
Exhaustion	7	1,344	.26	.10	.07	.31	.12	.09	(0.20, 0.43)	(0.19, 0.44)	47.47
Identity separation	10	12,280	.38	.08	.07	.49	.10	.09	( 0.42, 0.56)	( 0.36, 0.61)	17.73
Negative affect	5	1,465	.28	.11	.09	.34	.13	.11	(0.18, 0.50)	(0.17, 0.51)	26.84
Positive affect	7	1,627	−.39	.08	.06	−.47	.10	.07	(−0.56, −0.38)	(−0.57, −0.37)	51.13
Self-efficacy	7	1,545	−.21	.07	.00	−.26	.08	.00	(−0.33, −0.19)	(−0.26, −0.26)	100.15
Work authenticity	3	14,093	−.26	.01	.00	−.36	.02	.00	(−0.41, −0.32)	(−0.36, −0.36)	337.60
Work withdrawal											
Deviant behavior	4	905	.18	.13	.11	.23	.17	.15	(−0.04, 0.51)	(−0.01, 0.48)	24.52
Turnover intentions	25	5,115	.28	.10	.07	.34	.12	.09	(0.29, 0.39)	(0.23, 0.46)	46.34

*Note. k* = number of independent samples; *N* = cumulative sample size; $\bar{r}$ = mean observed correlation; $SD_{r}$ = observed standard deviation of r ; $SD_{res}$ = residual standard deviation of r ; $\bar{\rho }$ = mean true-score correlation; $SD_{r_{c}}$ = observed standard deviation of corrected correlations ( $r_{c}$ ); $SD_{\rho }$ = residual standard deviation of ρ ; CI = confidence interval around $\bar{\rho }$ ; CR = credibility interval around $\bar{\rho }$ ; % Var = variance attributable to sampling error and statistical artifacts. Correlations corrected using artifact distributions. Please note that the calculation of the presented 80 % credibility intervals (CRs) is based on *SD*_ρ_. Thus, in case *SD*_ρ_ is zero (i.e., there is no uncertainty in the population that cannot be attributed to artefactual variance), the CR is equivalent to the calculated value for . In these cases, we report the 95 % CI as a proxy for the CR.

Regarding *behavioral coping*, stereotype threat was positively related to both dysfunctional (ρ = .24) and functional behavioral coping (ρ = .15). Thus, here, our a priori assumptions were only partially supported, as we had expected a negative association with functional behavioral coping. Concerning *career-related* variables, stereotype threat was consistent with expectations negatively related to career aspirations (ρ = –.25) but was unrelated to career opportunities. As for *job attitudes* and *performance*, consistent with our a-priori expectations, stereotype threat was negatively related to organizational commitment (ρ = –.30), job satisfaction (ρ = –.34), job engagement (ρ = –.29), and job performance (ρ = –.35). In contrast, stereotype threat was not significantly related to organizational citizenship behavior (ρ = .16).

Concerning *well-being*, stereotype threat was positively related to exhaustion (ρ = .31), identity separation (ρ = .49), and negative affect (ρ = .34), and negatively related to positive affect (ρ = –.47), self-efficacy (ρ = –.26), and work authenticity (ρ = –.36). Thus, our hypotheses on stereotype threat and occupational well-being outcomes were supported. Finally, regarding *withdrawal behaviors*, stereotype threat was positively related to turnover intentions (ρ = .34), whereas it was not significantly related to deviant behavior (ρ = .23), partly supporting our a-priori expectations. Forest and funnel plots for relationships with *k* > 10 are reported in online supplemental material (OSM) S1.

### Moderator Analyses

Following best practice recommendations (Borenstein et al., 2021), and in line with common practice (e.g., Rudolph et al., 2017), we only conducted moderator analyses for constructs represented in at least *k* = 10 studies in the focal analyses (i.e., turnover intentions, job satisfaction, identity separation, job engagement, organizational commitment, functional behavioral coping, job performance, and career aspirations). Results are summarized below with detailed results of the moderator analyses presented in OSM S2.

Regarding the classification of stereotype threat (stereotype threat vs. stigma consciousness), there were no significant moderation effects. Three relationships were not significant when only considering studies assessing stigma consciousness (i.e., relationships with functional behavioral coping, job engagement, and job performance). However, these relationships all depended on a small number of samples, resulting in larger CIs.

Regarding stigmatized group (i.e., females vs. older workers), the negative relationships between stereotype threat and career aspirations, job satisfaction, and job engagement were stronger for older workers as the stereotyped group than for females as the stereotyped group. Specifically, the CI for the stereotype threat–career aspirations relationship in studies with females being stereotyped (–0.23, –0.05) did not overlap with the CI for studies with older workers being stereotyped (–0.34, –0.34). In addition, the CI for the stereotype threat-job satisfaction relationship in studies with females being stereotyped (–0.37, –0.16) did not overlap with the CI for studies with older workers being stereotyped (–0.59, –0.44). Finally, the CI for the stereotype threat-job engagement relationship in studies with females being stereotyped (–0.24, –0.10) did not overlap with the CI for studies with older workers being stereotyped (–0.38, –0.28). There were no significant moderation effects of the stigmatized group for the other potential consequences of stereotype threat.

### Sensitivity Analyses

Detailed results of these sensitivity analyses are presented in the OSM S3. We identified potentially influential cases by calculating Cook’s distances (values higher than 0.45 potentially influential) and standardized residuals (values ± 2.24 potentially influential). Based on these two parameters, we recalculated the meta-analyses excluding potentially influential cases (see OSM S4). There were no changes in the significance of the relationships when influential cases were excluded. Results of cumulative meta-analyses (see OSM S3) indicated that studies with lower precision (i.e., smaller *N*) caused slight “drift” in our meta-analytic estimate for identity separation (i.e., toward lower overall meta-analytic estimate), and job engagement (i.e., slight drift toward higher overall meta-analytic estimate).

### Test of Publication Bias

Egger’s tests for funnel plot asymmetry were nonsignificant for the eight constructs represented in at least *k* = 10 studies, suggesting that no publication bias was present (see results in OSM S5). Regarding the trim-and-fill procedure (see OSM S5), the null hypothesis that no studies were missing in the funnel plot was rejected for organizational commitment (*p* = .016), indicating some degree of publication bias. In a model including the original plus the imputed data of five additional studies, the $\bar{r}$ was −0.22 (as compared with −0.25 in our focal analyses), as is shown in the funnel plot in OSM S5. In addition, moderator analyses revealed that there was no significant moderation effect for publication status (i.e., published vs. unpublished; see OSM S2). Finally, we introduced publication year as a continuous moderator and specified meta-regression models. Publication year was found to moderate the strength of the negative relationship between stereotype threat and organizational commitment (*B* = 0.02), job satisfaction (*B* = 0.01), and job performance (*B* = −0.01). These results suggest that the negative relationship between stereotype threat and organizational commitment and job satisfaction decreased as a function of publication year, while the negative association between stereotype threat and job performance increased as a function of publication year. In addition, the relationship between stereotype threat and functional behavioral coping became more positive as a function of publication year (*B* = 0.02). These analyses are displayed in Figure S2 in OSM2.

### Quality Assessment

**Figure 2.**
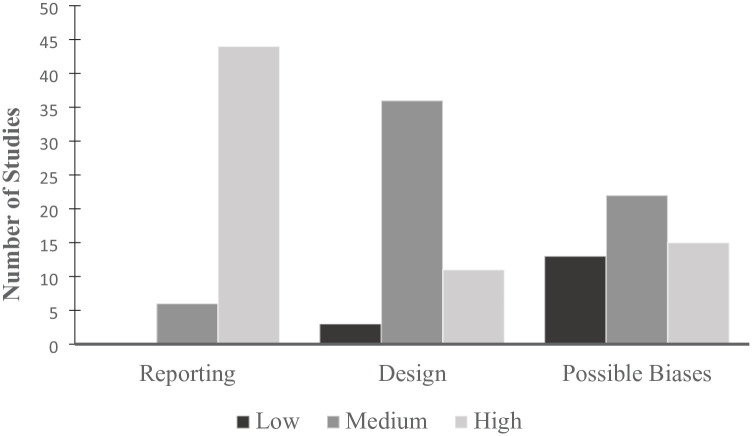
Quality Assessment: Level of Study Quality Across Three Quality Domains.

Across the three quality subcategories (i.e., reporting, design, and possible biases), most contributing studies were classified as medium to high quality (Figure 2). A more in-depth examination of each of the 17 criterion revealed that, while six of the AXIS criteria (i.e., clear aims/objectives, appropriate study design, appropriate sample frame, appropriate measures, clear significance parameters, and justified conclusions) were met by the included studies (90% and above), three of the assessment criteria were met by less than half of the studies evaluated. Specifically, only 14% of studies provided clear justification of the sample size (e.g., conducted an a priori power analysis), with most relying on samples available to them. In addition, 71% of studies failed to acknowledge whether they obtained ethical approval, whereas 63% of studies did not explicitly state whether there were any conflicts of interest to disclose (excluding unpublished theses for which this question was deemed nonapplicable, assigned as one). Taken together, although most of the studies were of medium to high quality across the three subcategories, there were common limitations in study design that should be taken into consideration when evaluating this meta-analysis. We introduced study quality as a continuous moderator and specified meta-regression models. Study quality moderated the strength of the negative relationship between stereotype threat and career aspirations, such that the association was stronger in studies with higher quality (*B* = −0.02). To check whether study quality might have improved over the years, we checked the association between study quality and publication year. Study quality was not significantly correlated with publication year (*r* = −0.02, *p* = .870). We additionally checked the relationship between the two constructs in regression analyses. We first fitted a simple linear regression model. In this model, publication year was not a significant predictor of study quality (*B* = −0.01, *p* = .870). To explore potential nonlinear effects, we extended the model by including a quadratic term. Neither the linear term (*B* = 0.00, *p* = .536) nor the quadratic term (*B* = 0.00, *p* = .536) significantly predicted study quality.

## Discussion

### Summary and Interpretation of Findings

This meta-analysis provides the first systematic integration of extant literature on the potential effects of stereotype threat in workplace settings, focusing on associations with various work-related outcomes. By integrating data from 61 independent data sets comprising more than 40,000 participants, results revealed significant relationships in the expected directions for nearly all outcomes examined. Specifically, stereotype threat was positively related to dysfunctional behavioral coping although, surprisingly, also positively related to functional behavioral coping (more on this below). Stereotype threat was negatively related to career aspirations, organizational commitment, job satisfaction, job engagement, job performance, positive affect, self-efficacy, and work authenticity. Furthermore, stereotype threat was positively associated with exhaustion, identity separation, negative affect, and turnover intentions. Using Bosco et al.’s (2015) correlational effect size benchmarks, most of these relationships were of medium effect size, with occasionally slightly larger effects. These findings indicate a consistent association between stereotype threat and a broad range of important work-related outcomes. However, the cross-sectional nature of most included studies limits the ability to draw causal conclusions.

Regarding moderation analyses, the stigmatized group (i.e., female gender vs. older age) moderated relationships between stereotype threat and certain workplace outcomes. Specifically, the negative relationships between stereotype threat and career aspirations, job satisfaction, and job engagement were more pronounced for older workers than women. This differential effect might be attributed to differences in the negativity of age- versus gender-based stereotypes. Older employees are portrayed as less adaptable, less competent with new technologies, and more resistant to change (e.g., Posthuma & Campion, 2009; see also Henry et al., 2023). Such stereotypes can lead to heightened levels of stereotype threat for older workers, thereby exacerbating negative job attitudes and reducing engagement. In contrast, while gender-based stereotype threat is also impactful, it is possible that the societal and organizational efforts to address gender inequality may have mitigated some of its adverse effects. Regardless, these findings underscore the need for tailored interventions to address the unique challenges faced by different stereotyped groups within the workplace.

Interestingly, our meta-analysis revealed that the relationships between stereotype threat and related constructs with certain workplace outcomes have changed in magnitude in more recent research. Specifically, the pattern of results observed in this study suggests that the detrimental effects of stereotype threat on organizational commitment and job satisfaction may be diminishing over time (the relationship between stereotype threat and job performance shows a slight increase in negativity over time, but this relationship should be interpreted with caution due to the small number of studies that measured performance). One possible explanation for this trend is the increasing use of positive coping mechanisms, as evidenced by the moderating effect of publication year on functional behavioral coping. Specifically, as publication year increases, individuals appear to be engaging more in constructive behavioral strategies to counteract the negative effects of stereotype threat, which may in turn buffer its impact on organizational commitment and job satisfaction.

Although a positive association between stereotype threat and adaptive coping behaviors was not anticipated, broader literature shows how stereotype threat can also evoke adaptive coping behaviors in certain contexts or individuals. For instance, stereotype threat might trigger a motivational response in some individuals, leading them to engage in adaptive coping behaviors to counteract the negative effects of the threat (Schmader et al., 2008; Yeung & von Hippel, 2008). This proactive coping can be seen as a form of resilience, where individuals use adaptive strategies to manage and mitigate the effects of stereotype threat. Relatedly, individuals who are aware of the potential for stereotype threat might develop adaptive coping strategies as a pre-emptive measure, such as seeking feedback, engaging in self-affirmation, or enhancing their skills to prove the stereotypes wrong (e.g., Cohen & Garcia, 2005). The presence of supportive environments or communities also influences how individuals cope with stereotype threat, with access to resources, such as mentorship, training, or peer support, linked to an increased likelihood of engaging in adaptive coping behaviors (e.g., Murphy et al., 2007). Alternatively, when the task at hand is highly valued or crucial to an individual’s goals, they might be more likely to adopt adaptive coping strategies to maintain performance and protect their self-esteem (Cohen & Sherman, 2014). The desire to succeed despite experiences of stereotype threat may drive individuals to use positive coping mechanisms.

Stereotype reactance may also explain the relationship between stereotype threat and adaptive coping behaviors. As discussed by Hoyt and Murphy (2016), stereotype reactance refers to the phenomenon where individuals who are aware of negative stereotypes about their social group may actively resist these stereotypes. This resistance can manifest in various ways, including increased motivation to disprove the stereotype or enhanced performance in stereotype-relevant tasks (e.g., Kray et al., 2001). Incorporating this perspective may help to explain why stereotype threat does not always lead to negative outcomes and, in some cases, may even result in adaptive behaviors. For instance, individuals facing stereotype threat may channel their awareness of the stereotype into a determination to succeed, thereby mitigating the stereotype’s potential harmful effects. This possibility aligns with the idea that stereotype threat is not a monolithic construct but one that can elicit a range of responses depending on the context, individual differences, and the resources available to the person experiencing it.

Finally, the increase in functional coping could reflect broader societal and organizational shifts toward recognizing and addressing stereotype threat. Over time, there has been growing awareness of the ways in which stereotype threat undermines performance and well-being, along with a corresponding emphasis on promoting resilience and adaptive strategies. As organizations adopt more inclusive practices and individuals become more adept at navigating stereotype-related challenges, the harmful effects of stereotype threat may be mitigated. In sum, the diminishing negative impact of stereotype threat on organizational commitment and job satisfaction, alongside the rise in functional behavioral coping, points to a shift toward more effective management of stereotype-related challenges in the workplace. Further research is needed to explore the potential mechanisms underlying these trends and to identify additional factors that contribute to the development of stereotype threat over time.

### Theoretical Implications

The significant relationships identified in this meta-analysis have theoretical implications. Our findings support and extend Steele’s (1997) theory of stereotype threat, highlighting its relevance in diverse workplace contexts. Nonetheless, these findings should be interpreted with caution due to the predominance of cross-sectional studies (and studies with incomplete panel designs) in the analysis.

The fact that recent meta-analyses (e.g., Picho-Kiroga et al., 2021; Warne, 2022) have questioned the performance decrements associated with stereotype threat does not undermine the importance of our findings. Rather, our study complements this body of work by extending the investigation of stereotype threat beyond the laboratory and into real-world work environments where the stakes and dynamics fundamentally differ. By doing so, we contribute to a more comprehensive understanding of how stereotype threat manifests and impacts individuals in their everyday work lives, regardless of its effects on specific performance tasks.

A further important goal of this meta-analysis was to determine whether stereotype threat, stigma consciousness, and meta-stereotyping, have similar relationships with the workplace outcomes included in our analysis. The results revealed no significant differences between stereotype threat and stigma consciousness with several important workplace outcomes. These findings suggest that, while stereotype threat and stigma consciousness are theoretically distinct constructs, their relationship with workplace outcomes may not be as empirically distinguishable as previously assumed. Stereotype threat is traditionally viewed as a situationally induced state, where individuals fear confirming negative stereotypes about their group in specific contexts (Steele, 1997). Collectively, the research demonstrating the disengagement consequences of stereotype threat provides further support for Steele’s (1997) original theorizing. In contrast, stigma consciousness is conceptualized as a chronic, individual difference variable, reflecting an ongoing awareness of, and sensitivity to, stereotypes about one’s group (Pinel, 1999). However, our meta-analysis revealed that the magnitude of their relations with work-related attitudes and behaviors was remarkably similar.

This overlap in relations with key outcomes suggests that both constructs may tap into common underlying psychological processes, such as heightened stress or vigilance in environments where negative stereotypes are salient. The similarity in their effects raises important questions about whether these constructs are truly distinct in practice, or whether they represent different facets of a more general sensitivity to being stereotyped. Future research should aim to further disentangle these constructs by examining the specific conditions under which stereotype threat and stigma consciousness may produce unique outcomes, as well as exploring potential moderating variables that might explain when and why their effects converge or diverge. Alternatively, the nonsignificant moderation effects we observed when comparing stereotype threat to stigma consciousness may mean that these constructs are interchangeable. Unfortunately, the limited number of studies examining meta-stereotyping did not allow us to compare it to stereotype threat and stigma consciousness and its relationship with workplace outcomes.

A critical distinction in stereotype threat research lies between experimental studies, which manipulate the presence of stereotype threat, and correlational studies, which measure the chronic experiences of stereotype threat in real-world settings. Our meta-analysis focuses on the latter, seeking to assess the relevance of stereotype threat in everyday workplace environments. Experimental studies have provided evidence for the causal effects of stereotype threat, often demonstrating that individuals under threat perform worse on tasks that are relevant to the stereotype. These studies typically involve short-term manipulations in controlled settings, where the presence of stereotype threat can be isolated and measured with precision. However, while these studies are invaluable for understanding the immediate causal effects of stereotype threat, they do not capture the ongoing, chronic nature of stereotype threat as it is experienced in the workplace. In contrast, correlational studies measure the chronic presence of stereotype threat and their relations with longer-term outcomes, such as job satisfaction, career aspirations, and turnover intentions. The relations observed in these studies may differ in magnitude from those seen in experimental research, as they reflect the cumulative impact of stereotype threat over time rather than the immediate consequences of a single threat-inducing situation (Kalokerinos et al., 2014).

The differences between experimental and correlational research have significant implications for the broader theory of stereotype threat. While experimental studies provide clear evidence of the potential for stereotype threat to impair performance, correlational studies offer insights into its broader relationships with individuals’ work experiences and career trajectories. Together, these findings suggest that stereotype threat is not merely a performance-related issue but also a significant factor influencing the long-term success and well-being of employees.

### Practical Implications

The relationship between stereotype threat and workplace outcomes suggests that organizations should prioritize interventions aimed at addressing stereotype threat to foster more inclusive work environments. Effective interventions, such as stereotype awareness training and affirmation interventions, have been shown to reduce the impact of stereotype threat on performance (Liu et al., 2021). Future research should determine whether these interventions could be imported into the workplace. For example, employees could be taught how to reframe experiences that lead to stereotype threat to hopefully mitigate the negative consequences, or possibly reduce feelings of stereotype threat directly.

Recognizing that stereotype threat can, under certain conditions, lead to positive coping mechanisms, organizations might consider strategies that promote these adaptive responses. For example, training programs that emphasize resilience, self-efficacy, and stereotype management could help employees channel the pressure of stereotype threat into positive outcomes. Future research should investigate these mechanisms further to understand precisely when, why and how the experience of stereotype threat can trigger an adaptive coping response and use this knowledge to develop more effective interventions that mitigate the adverse effects of stereotype threat in the workplace.

### Limitations and Future Research Directions

Despite the strengths of this meta-analysis, several limitations should be acknowledged. First, while our findings clearly demonstrate significant relationships between stereotype threat and adverse workplace outcomes such as reduced job satisfaction, organizational commitment, and job engagement, as discussed, the cross-sectional nature of nearly all included studies limits our ability to establish causality. It is plausible that employees who already harbor negative job attitudes may be more likely to perceive or experience stereotype threat, as their dissatisfaction and disengagement could heighten their sensitivity to negative stereotypes and discriminatory cues in the workplace. This bidirectional relationship suggests that, while stereotype threat can contribute to negative work outcomes, pre-existing negative work outcomes might also exacerbate perceptions of stereotype threat (see Coulon et al., 2024, for preliminary data supporting this possibility). Future research using longitudinal and experimental designs is needed to determine the causal pathways underlying the relationships between stereotype threat and workplace outcomes. Another limitation of our meta-analysis is that most of the outcome variables were based on self-report measures, which can introduce biases such as common method variance (Podsakoff et al., 2003). Future research should incorporate objective measures and behavioral data to provide a more comprehensive and accurate assessment of the impact of stereotype threat on workplace outcomes. Finally, although our analysis indicated that publication bias was not a significant issue for most of the constructs examined, it is important to note that the findings with organizational commitment did show some degree of publication bias. The trim-and-fill analysis suggested that this bias slightly impacted the overall effect size, though the relationship between stereotype threat and organizational commitment remained significant even after adjusting for potentially missing studies. It is also worth noting that publication bias is an unlikely explanation because we included unpublished data sets that were gathered through listservs and by emailing researchers directly who have published in this area.

Our meta-analysis provides an opportunity to reflect on the strengths and limitations of the methodological approaches and to offer recommendations for future research. As discussed, the frequent reliance on cross-sectional designs and self-reported outcome measures limits the ability to draw causal conclusions and raises concerns about common method bias. It should be noted, however, that longitudinal panel designs and capturing objective outcome measures (e.g., actual turnover instead of turnover intentions) can be extremely difficult to execute in practice, hence the reliance on correlational, self-report designs. In addition, innovative methods such as diary studies and experience sampling methods capture the dynamic nature of stereotype threat as it unfolds in real-world settings. These approaches allow for the examination of within-person variations in stereotype threat experiences, offering valuable insights into how individuals cope with and respond to stereotype threat on a day-to-day basis. By capturing these fluctuations, researchers can gain a deeper understanding of the temporal dynamics of stereotype threat and its impact on workplace behavior and attitudes. Such approaches (i.e., longitudinal designs and objective measurement of outcome variables) would provide a more robust understanding of whether stereotype threat has a causal influence on workplace outcomes over time and help to mitigate the potential biases associated with self-reported data.

In addition, although we have assumed in this review that manipulations of stereotype threat do not capture the same aspects as chronic feelings of stereotype threat in the workplace that remains an untested empirical assumption. Research could attempt to manipulate stereotype threat in the workplace (e.g., through reminders of important prior experiences), to assess whether the outcomes are similar when stereotype threat is manipulated vs. measured. For example, von Hippel et al. (2017) found that male child protection workers who engaged in an upward social comparison with a colleague who successfully handled a concern about sexual abuse in a feminine manner reported greater intentions to quit their jobs compared with employees who did not engage in the upward comparison.

Related to the measurement of stereotype threat, a notable limitation of many stereotype threat scales used in workplace research is the frequent omission of the “worry” component, which is central to the experience of stereotype threat. For instance, a typical item in these scales might be “My colleagues think I’m less committed because of my age,” rather than “I worry my colleagues think I’m less committed because of my age.” This distinction may be crucial, as the “worry” aspect directly taps into the anxiety and concern that are key features of stereotype threat. The absence of this component in many scales can be traced back to the original conceptualization of stereotype threat by Steele and Aronson (1995). In their work, the focus was on the performance implications of stereotype threat, and the items used to measure it did not explicitly include the term “worry.” As a result, many subsequent adaptations of these scales for workplace settings have retained this structure, potentially overlooking the full emotional impact of stereotype threat. In summary, the lack of emphasis on “worry” in many of the scales to measure stereotype threat in the workplace may lead to an incomplete understanding of how stereotype threat operates in real-world environments, where the ongoing concern about confirming negative stereotypes can significantly affect an individual’s work experience. Future research should consider revising and expanding existing stereotype threat scales to explicitly include items that capture the worry component. By doing so, researchers can gain a more comprehensive understanding of the psychological mechanisms underlying stereotype threat in the workplace and better assess its impact on employee outcomes.

## Concluding Remarks

In conclusion, this meta-analysis provides robust evidence for the significant impact of stereotype threat on a wide range of workplace outcomes. Our meta-analysis also revealed that the effects of stereotype threat were stronger for age-related stereotypes compared with gender-related stereotypes for certain workplace outcomes, and that the strength of these effects are changing with the passage of time depending on the outcome studied. These observations warrant further investigation, particularly through longitudinal studies, to confirm these patterns. The underlying reasons for these differences remain unknown, highlighting the need for further research to explore the mechanisms driving these varying impacts. By highlighting these relationships, we hope to inform organizational policies and interventions aimed at reducing stereotype threat and promoting a more inclusive workplace environment.

## Supplemental Material

sj-docx-1-psp-10.1177_01461672241297884 – Supplemental material for Stereotype Threat at Work: A Meta-Analysis Supplemental material, sj-docx-1-psp-10.1177_01461672241297884 for Stereotype Threat at Work: A Meta-Analysis by Courtney von Hippel, Clara Kühner, Sarah P. Coundouris, Amy Lim, Julie D. Henry and Hannes Zacher in Personality and Social Psychology Bulletin

## Notes

1. We excluded an effect size for the relationship between stereotype threat and turnover intentions from the dissertation by Membere (2018), because the direction of the relationship was not clear (i.e., contradictory information in the correlation table and in the text) and we were unable to reach the author.

2. Two studies considered two types of stereotype threat in partially overlapping subsamples. Specifically, the von Hippel data set and the Manzi, Sorgente et al., (2021) study reported results for age-based stereotype threat for the whole sample and results on gender-based stereotype threat for the subsample of females only. To ensure independency of effect sizes, we excluded the female subsamples from the main analyses of these two studies and retained only the full sample data for age-based stereotype threat. However, both subsamples were included in the moderator analyses, where we differentiated between age- and gender-based stereotype threat.

3. In the preregistration, we planned to impute missing reliabilities with average reliabilities of a specific measure from our meta-analytic database. However, since we applied artifact distribution meta-analyses, no such imputation was required (Wiernik & Dahlke, 2020)
