# Supplementary material for: Stereotype Threat at Work: A Meta-Analysis
Source: Pers Soc Psychol Bull. 2024 Dec 13;52(4):927–48. doi: 10.1177/01461672241297884 (PMC12949755; doi:10.1177/01461672241297884)
Supplement: sj-docx-1-psp-10.1177_01461672241297884 – Supplemental material for Stereotype Threat at Work: A Meta-Analysis [file sj-docx-1-psp-10.1177_01461672241297884.docx]

**Table S2**

*Exploratory Moderator Analyses*

| **FUNCTIONAL BEHAVIORAL COPING** | | | | | | | | | | | | | | | | | |
| --- | --- | --- | --- | --- | --- | --- | --- | --- | --- | --- | --- | --- | --- | --- | --- | --- | --- |
|  |  | ***k*** | ***N*** | $\overline{\boldsymbol{r}}$ | | $\boldsymbol{S}\boldsymbol{D}_{\boldsymbol{r}}$ | | $\boldsymbol{S}\boldsymbol{D}_{\boldsymbol{res}}$ | | $\overline{\boldsymbol{\rho}}$ | | $\boldsymbol{S}\boldsymbol{D}_{\boldsymbol{r}_{\boldsymbol{c}}}$ | | $\boldsymbol{S}\boldsymbol{D}_{\boldsymbol{\rho}}$ | **95% CI** | | **80% CR** |
|  | Overall | 15 | 3,785 | .12 | | .21 | | .20 | | .15 | | .25 | | .24 | [ 0.01, 0.28] | | [−0.18, 0.47] |
| **Classification** | Stereotype threat | 12 | 2,663 | .16 | | .24 | | .24 | | .19 | | .30 | | .29 | [−0.00,  0.38] | | [−0.20,  0.58] |
|  | Stigma consc. | 4 | 1,219 | .07 | | .11 | | .09 | | .08 | | .13 | | .11 | [−0.13,  0.29] | | [−0.10,  0.27] |
| **Group** | Female gender | 3 | 1,085 | .13 | | .14 | | .13 | | .16 | | .18 | | .16 | [−0.28,  0.60] | | [−0.15,  0.47] |
|  | Older age | 3 | 247 | .22 | | .25 | | .23 | | .26 | | .31 | | .28 | [−0.50,  1.03] | | [−0.26,  0.79] |
| **Status** | Published | 12 | 2,907 | .16 | | .18 | | .16 | | .20 | | .21 | | .20 | [ 0.06,  0.33] | | [−0.08,  0.47] |
|  | Unpublished | 3 | 878 | −.02 | | .28 | | .27 | | −.02 | | .34 | | .33 | [−0.87,  0.83] | | [−0.65,  0.61] |
|  |  |  |  | | **Estimate** | | ***SE*** | | ***Z*** | | ***p*** | | **95% CI** | | | **Residual Heterogeneity** | |
| **Year** | Intercept  Year |  |  | | -37.40  0.02 | | 16.35  0.01 | | -2.29  2.29 | | 0.022  0.022 | | [-69.45,-5.35]  [ 0.00, 0.03] | | | QE(df = 12) = 92.6480,  p < .001; I^2^ = 83.96% | |
| **Quality** | Intercept  Quality |  |  | | 0.67  -0.04 | | 0.38  0.03 | | 1.76  -1.44 | | 0.079  0.151 | | [-0.07, 1.41]  [-0.10, 0.02] | | | QE(df = 11) = 113.4238,  p < .001; I^2^ = 87.58% | |
| **CAREER ASPIRATIONS** | | | | | | | | | | | | | | | | | |
|  |  | ***k*** | ***N*** | $\overline{\boldsymbol{r}}$ | | $\boldsymbol{S}\boldsymbol{D}_{\boldsymbol{r}}$ | | $\boldsymbol{S}\boldsymbol{D}_{\boldsymbol{res}}$ | | $\overline{\boldsymbol{\rho}}$ | | $\boldsymbol{S}\boldsymbol{D}_{\boldsymbol{r}_{\boldsymbol{c}}}$ | | $\boldsymbol{S}\boldsymbol{D}_{\boldsymbol{\rho}}$ | **95% CI** | | **80% CR** |
|  | Overall | 10 | 11,568 | −.19 | | .09 | | .09 | | −.25 | | .12 | | .12 | [−0.34, −0.17] | | [−0.41, −0.09] |
| **Group** | Female gender | 6 | 4,839 | −.11 | | .06 | | .05 | | −.14 | | .09 | | .07 | [−0.23, −0.05] | | [−0.25, −0.04] |
|  | Older age | 3 | 6,499 | −.26 | | .00 | | .00 | | −.34 | | .00 | | .00 | [−0.34, −0.34] | | [−0.34, −0.34] |
| **Status** | Published | 5 | 3,212 | −.24 | | .07 | | .06 | | −.32 | | .09 | | .08 | [−0.43, −0.20] | | [−0.43, −0.20] |
|  | Unpublished | 5 | 8,356 | −.17 | | .10 | | .10 | | −.23 | | .13 | | .13 | [−0.39, −0.06] | | [−0.43, −0.03] |
|  |  |  |  | | **Estimate** | | ***SE*** | | ***Z*** | | ***p*** | | **95% CI** | | | **Residual Heterogeneity** | |
| **Year** | Intercept  Year |  |  | | -3.36  0.00 | | 25.15  0.01 | | -0.13  0.12 | | 0.894  0.901 | | [-52.64, 45.93]  [ -0.02, 0.03] | | | QE(df = 6) = 27.5250,  p < .01; I^2^ = 56.47% | |
| **Quality** | Intercept  Quality |  |  | | 0.10  -0.02 | | 0.12  0.01 | | 0.88  -2.66 | | 0.381  0.008 | | [-0.13, 0.34]  [-0.04, -0.01] | | | QE(df = 6) = 15.7407,  p < .05; I^2^ = 37.53% | |
| **ORGANIZATIONAL COMMITMENT** | | | | | | | | | | | | | | | | | |
|  |  | ***k*** | ***N*** | $\overline{\boldsymbol{r}}$ | | $\boldsymbol{S}\boldsymbol{D}_{\boldsymbol{r}}$ | | $\boldsymbol{S}\boldsymbol{D}_{\boldsymbol{res}}$ | | $\overline{\boldsymbol{\rho}}$ | | $\boldsymbol{S}\boldsymbol{D}_{\boldsymbol{r}_{\boldsymbol{c}}}$ | | $\boldsymbol{S}\boldsymbol{D}_{\boldsymbol{\rho}}$ | **95% CI** | | **80% CR** |
|  | Overall | 17 | 17,608 | −.25 | | .09 | | .09 | | −.30 | | .11 | | .11 | [−0.36, −0.24] | | [−0.45, −0.16] |
| **Classification** | Stereotype threat | 17 | 20,482 | −.21 | | .09 | | .08 | | −.26 | | .11 | | .10 | [−0.32, −0.21] | | [−0.39, −0.13] |
|  | Stigma consc. | 5 | 740 | −.29 | | .10 | | .06 | | −.36 | | .12 | | .08 | [−0.51, −0.21] | | [−0.48, −0.24] |
| **Group** | Female gender | 7 | 4,688 | −.14 | | .11 | | .11 | | −.17 | | .14 | | .13 | [−0.30, −0.04] | | [−0.36,  0.02] |
|  | Older age | 9 | 16,021 | −.24 | | .09 | | .08 | | −.30 | | .11 | | .10 | [−0.38, −0.22] | | [−0.44, −0.16] |
| **Status** | Published | 12 | 16,502 | −.24 | | .08 | | .08 | | −.30 | | .10 | | .10 | [−0.36, −0.23] | | [−0.43, −0.17] |
|  | Unpublished | 5 | 1,106 | −.30 | | .20 | | .19 | | −.36 | | .25 | | .24 | [−0.68, −0.05] | | [−0.73, 0.00] |
|  |  |  |  | | **Estimate** | | ***SE*** | | ***Z*** | | ***p*** | | **95% CI** | | | **Residual Heterogeneity** | |
| **Year** | Intercept  Year |  |  | | -35.81  0.02 | | 15.02  0.01 | | -2.38  2.36 | | 0.017  0.018 | | [-65.25, -6.36]  [ 0.00, 0.03] | | | QE(df = 10) = 83.5519,  p < .001; I^2^ = 79.40% | |
| **Quality** | Intercept  Quality |  |  | | -0.48  0.02 | | 0.30  0.02 | | -1.61  0.74 | | 0.108  0.460 | | [-1.06, 0.10]  [-0.03, 0.06] | | | QE(df = 10) = 102.6093,  p < .001; I^2^ = 74.66% | |

| **JOB SATISFACTION** | | | | | | | | | | | | | | | | | |
| --- | --- | --- | --- | --- | --- | --- | --- | --- | --- | --- | --- | --- | --- | --- | --- | --- | --- |
|  |  | ***k*** | ***N*** | $\overline{\boldsymbol{r}}$ | | $\boldsymbol{S}\boldsymbol{D}_{\boldsymbol{r}}$ | | $\boldsymbol{S}\boldsymbol{D}_{\boldsymbol{res}}$ | | $\overline{\boldsymbol{\rho}}$ | | $\boldsymbol{S}\boldsymbol{D}_{\boldsymbol{r}_{\boldsymbol{c}}}$ | | $\boldsymbol{S}\boldsymbol{D}_{\boldsymbol{\rho}}$ | **95% CI** | | **80% CR** |
|  | Overall | 22 | 6,374 | −.28 | | .14 | | .13 | | −.34 | | .17 | | .15 | [−0.42, −0.27] | | [−0.54, −0.14] |
| **Classification** | Stereotype threat | 16 | 4,675 | −.30 | | .14 | | .12 | | −.37 | | .16 | | .15 | [−0.46, −0.28] | | [−0.57, −0.17] |
|  | Stigma consc. | 11 | 2,421 | −.23 | | .12 | | .10 | | −.28 | | .14 | | .12 | [−0.37, −0.18] | | [−0.44, −0.12] |
| **Group** | Female gender | 11 | 3,571 | −.22 | | .13 | | .11 | | −.27 | | .15 | | .14 | [−0.37, −0.16] | | [−0.45, −0.08] |
|  | Older age | 6 | 1,794 | −.42 | | .06 | | .03 | | −.51 | | .07 | | .04 | [−0.59, −0.44] | | [−0.57, −0.46] |
| **Status** | Published | 14 | 5,193 | −.26 | | .13 | | .12 | | −.32 | | .16 | | .15 | [−0.41, −0.22] | | [−0.52, −0.12] |
|  | Unpublished | 8 | 1,181 | −.37 | | .14 | | .12 | | −.45 | | .17 | | .14 | [−0.59, −0.30] | | [−0.65, −0.24] |
|  |  |  |  | | **Estimate** | | ***SE*** | | ***Z*** | | ***p*** | | **95% CI** | | | **Residual Heterogeneity** | |
| **Year** | Intercept  Year |  |  | | -29.27  0.01 | | 13.08  0.01 | | -2.24  2.22 | | 0.025  0.027 | | [-54.9, -3.64]  [ 0.00, 0.03] | | | QE(df = 14) = 74.1582,  p < .0001; I^2^ = 77.15% | |
| **Quality** | Intercept  Quality |  |  | | -0.22  -0.00 | | 0.19  0.01 | | -1.15  -0.25 | | 0.251  0.805 | | [-0.58, 0.15]  [-0.03, 0.02] | | | QE(df = 13) = 99.0120,  p < .001; I^2^ = 84.35% | |

| **JOB ENGAGEMENT** | | | | | | | | | | | | | | | | | | | | |
| --- | --- | --- | --- | --- | --- | --- | --- | --- | --- | --- | --- | --- | --- | --- | --- | --- | --- | --- | --- | --- |
|  |  | ***k*** | ***N*** | | $\overline{\boldsymbol{r}}$ | | | $\boldsymbol{S}\boldsymbol{D}_{\boldsymbol{r}}$ | | | $\boldsymbol{S}\boldsymbol{D}_{\boldsymbol{res}}$ | | $\overline{\boldsymbol{\rho}}$ | | $\boldsymbol{S}\boldsymbol{D}_{\boldsymbol{r}_{\boldsymbol{c}}}$ | | $\boldsymbol{S}\boldsymbol{D}_{\boldsymbol{\rho}}$ | **95% CI** | | **80% CR** |
|  | Overall | 15 | 18,498 | | −.23 | | | .07 | | | .07 | | −.29 | | .10 | | .09 | [−0.34, −0.24] | | [−0.41, −0.17] |
| **Classification** | Stereotype threat | 13 | 18,083 | | −.23 | | | .08 | | | .07 | | −.29 | | .10 | | .09 | [−0.35, −0.23] | | [−0.41, −0.17] |
|  | Stigma consc. | 3 | 624 | | −.17 | | | .08 | | | .04 | | −.22 | | .10 | | .05 | [−0.47, 0.04] | | [−0.31, −0.12] |
| **Group** | Female gender | 5 | 4,587 | | −.13 | | | .04 | | | .03 | | −.17 | | .06 | | .03 | [−0.24, −0.10] | | [−0.22, −0.12] |
|  | Older age | 8 | 13,641 | | −.26 | | | .05 | | | .03 | | −.33 | | .06 | | .05 | [−0.38, −0.28] | | [−0.40, −0.27] |
| **Status** | Published | 11 | 10,237 | | −.26 | | | .06 | | | .05 | | −.33 | | .08 | | .07 | [−0.39, −0.28] | | [−0.43, −0.24] |
|  | Unpublished | 4 | 8,261 | | −.19 | | | .08 | | | .07 | | −.24 | | .10 | | .09 | [−0.39, −0.09] | | [−0.39, −0.09] |
|  |  |  |  | | | **Estimate** | | | ***SE*** | | | ***Z*** | | ***p*** | | **95% CI** | | | **Residual Heterogeneity** | |
| **Year** | Intercept  Year |  |  | | | 32.24  -0.02 | | | 20.30  0.01 | | | 1.59  -1.60 | | 0.112  0.110 | | [-7.55, 72.04]  [-0.04, 0.00] | | | QE(df = 10) = 39.2778,  p < .001; I^2^ = 62.47% | |
| **Quality** | Intercept  Quality |  |  | | | -0.45  0.01 | | | 0.25  0.02 | | | -1.79  0.78 | | 0.07  0.44 | | [-0.94, 0.04]  [-0.02, 0.05] | | | QE(df = 9) = 37.3152,  p < .001; I^2^ = 59.10% | |
| **JOB PERFORMANCE** | | | | | | | | | | | | | | | | | | | | |
|  |  | ***k*** | ***N*** | | $\overline{\boldsymbol{r}}$ | | | $\boldsymbol{S}\boldsymbol{D}_{\boldsymbol{r}}$ | | | $\boldsymbol{S}\boldsymbol{D}_{\boldsymbol{res}}$ | | $\overline{\boldsymbol{\rho}}$ | | $\boldsymbol{S}\boldsymbol{D}_{\boldsymbol{r}_{\boldsymbol{c}}}$ | | $\boldsymbol{S}\boldsymbol{D}_{\boldsymbol{\rho}}$ | **95% CI** | | **80% CR** |
|  | Overall | 12 | 19,972 | | −.28 | | | .08 | | | .07 | | −.35 | | .10 | | .09 | [−0.41, −0.29] | | [−0.48, −0.23] |
| **Classification** | Stereotype threat | 12 | 23,104 | | −.24 | | | .14 | | | .14 | | −.30 | | .17 | | .17 | [−0.41, −0.19] | | [−0.53, −0.06] |
|  | Stigma consc. | 3 | 499 | | −.06 | | | .18 | | | .16 | | −.08 | | .22 | | .20 | [−0.62, 0.47] | | [−0.45, 0.29] |
| **Group** | Female gender | 4 | 7,614 | | −.16 | | | .22 | | | .22 | | −.20 | | .28 | | .28 | [−0.65, 0.24] | | [−0.66, 0.25] |
|  | Older age | 3 | 13,519 | | −.28 | | | .06 | | | .06 | | −.35 | | .08 | | .08 | [−0.54, −0.15] | | [−0.49, −0.20] |
| **Status** | Published | 5 | 10,057 | | −.23 | | | .06 | | | .05 | | −.29 | | .07 | | .06 | [−0.38, −0.20] | | [−0.39, −0.19] |
|  | Unpublished | 7 | 9,915 | | −.34 | | | .06 | | | .05 | | −.42 | | .08 | | .07 | [−0.49, −0.35] | | [−0.51, −0.32] |
|  |  |  |  | | | **Estimate** | | | ***SE*** | | | ***Z*** | | ***p*** | | **95% CI** | | | **Residual Heterogeneity** | |
| **Year** | Intercept  Year |  |  | | | 25.30  -0.01 | | | 9.28  0.00 | | | 2.73  -2.74 | | 0.006  0.006 | | [7.10, 43.50]  [-0.02, -0.00] | | | QE(df = 6) = 33.3538,  p < .001; I^2^ = 51.90% | |
| **Quality** | Intercept  Quality |  |  | | | -0.34  0.01 | | | 0.21  0.02 | | | -1.65  0.43 | | 0.099  0.668 | | [-0.74, 0.06]  [-0.02, 0.04] | | | QE(df = 6) = 58.9267,  p < .001; I^2^ = 68.41% | |
| **IDENTITY SEPARATION** | | | | | | | | | | | | | | | | | | | | |
|  |  | ***k*** | ***N*** | $\overline{\boldsymbol{r}}$ | | | $\boldsymbol{S}\boldsymbol{D}_{\boldsymbol{r}}$ | | | $\boldsymbol{S}\boldsymbol{D}_{\boldsymbol{res}}$ | | | $\overline{\boldsymbol{\rho}}$ | | $\boldsymbol{S}\boldsymbol{D}_{\boldsymbol{r}_{\boldsymbol{c}}}$ | | $\boldsymbol{S}\boldsymbol{D}_{\boldsymbol{\rho}}$ | **95% CI** | | **80% CR** |
|  | Overall | 10 | 12,280 | .38 | | | .08 | | | .07 | | | .49 | | .10 | | .09 | [ 0.42, 0.56] | | [ 0.36, 0.61] |
| **Status** | Published | 6 | 3,882 | .40 | | | .07 | | | .06 | | | .51 | | .09 | | .07 | [ 0.42, 0.60] | | [ 0.40, 0.62] |
|  | Unpublished | 4 | 8,398 | .37 | | | .09 | | | .08 | | | .48 | | .11 | | .11 | [ 0.30, 0.65] | | [ 0.30, 0.65] |
|  |  |  |  | | **Estimate** | | | | ***SE*** | | | ***Z*** | | ***p*** | | **95% CI** | | | **Residual Heterogeneity** | |
| **Year** | Intercept  Year |  |  | | 19.16  -0.01 | | | | 16.82  0.01 | | | 1.14  -1.11 | | 0.255  0.265 | | [-13.8, 52.1]  [-0.03, 0.01] | | | QE(df = 4) = 18.3676,  p < .05; I^2^ = 58.88% | |
| **Quality** | Intercept  Quality |  |  | | 0.20  0.01 | | | | 0.16  0.01 | | | 1.23  1.18 | | 0.217  0.238 | | [-0.12, 0.52]  [-0.01, 0.03] | | | QE(df = 4) = 18.6384,  p < .001; I^2^ = 61.33% | |

| **TURNOVER INTENTIONS** | | | | | | | | | | | | | | | | | |
| --- | --- | --- | --- | --- | --- | --- | --- | --- | --- | --- | --- | --- | --- | --- | --- | --- | --- |
|  |  | ***k*** | ***N*** | $\overline{\boldsymbol{r}}$ | | $\boldsymbol{S}\boldsymbol{D}_{\boldsymbol{r}}$ | | $\boldsymbol{S}\boldsymbol{D}_{\boldsymbol{res}}$ | | $\overline{\boldsymbol{\rho}}$ | | $\boldsymbol{S}\boldsymbol{D}_{\boldsymbol{r}_{\boldsymbol{c}}}$ | | $\boldsymbol{S}\boldsymbol{D}_{\boldsymbol{\rho}}$ | **95% CI** | | **80% CR** |
|  | Overall | 25 | 5,5115 | .28 | | .10 | | .07 | | .34 | | .12 | | .09 | [ 0.29, 0.39] | | [ 0.23, 0.46] |
| **Classification** | Stereotype threat | 20 | 3,853 | .30 | | .11 | | .09 | | .36 | | .14 | | .11 | [ 0.30, 0.43] | | [ 0.22, 0.50] |
|  | Stigma consc. | 9 | 1,816 | .25 | | .06 | | .00 | | .30 | | .08 | | .00 | [ 0.25, 0.36] | | [ 0.30, 0.30] |
| **Group** | Female gender | 13 | 2,439 | .27 | | .11 | | .09 | | .32 | | .14 | | .11 | [ 0.24, 0.41] | | [ 0.17, 0.47] |
|  | Older age | 8 | 2,064 | .30 | | .10 | | .07 | | .36 | | .12 | | .09 | [ 0.27, 0.46] | | [ 0.24, 0.49] |
| **Status** | Published | 15 | 3,543 | .26 | | .07 | | .02 | | .32 | | .08 | | .02 | [ 0.27, 0.36] | | [ 0.29, 0.34] |
|  | Unpublished | 10 | 1,572 | .33 | | .14 | | .12 | | .40 | | .17 | | .15 | [ 0.28, 0.52] | | [ 0.20, 0.60] |
|  |  |  |  | | **Estimate** | | ***SE*** | | ***Z*** | | ***p*** | | **95% CI** | | | **Residual Heterogeneity** | |
| **Year** | Intercept  Year |  |  | | 0.51  -0.00 | | 7.14  0.00 | | 0.07  -0.04 | | 0.943  0.972 | | [-13.48, 14.5]  [-0.01, 0.01] | | | QE(df = 16) = 26.0475,  p =.054; I^2^ = 29.67% | |
| **Quality** | Intercept  Quality |  |  | | 0.22  0.00 | | 0.08  0.01 | | 2.61  0.48 | | 0.009  0.628 | | [0.05, 0.38]  [-0.01, 0.02] | | | QE(df = 15) = 21.5221,  p = .121; I^2^ = 20.13% | |

**Figure S2**

*Moderation Effects of Publication Year*

| **(a) Functional Behavioral Coping**  **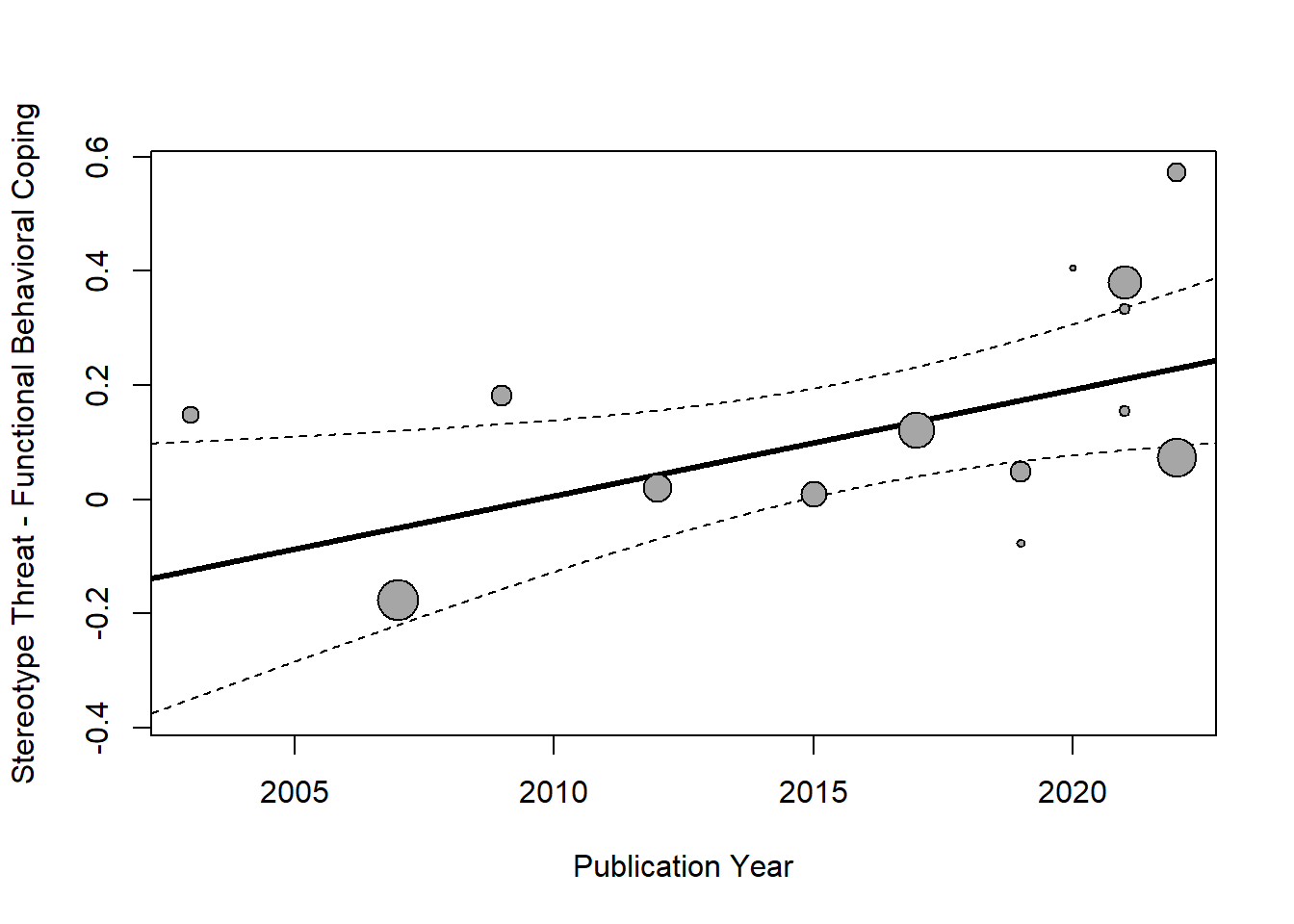** | **(b) Organizational Commitment**  **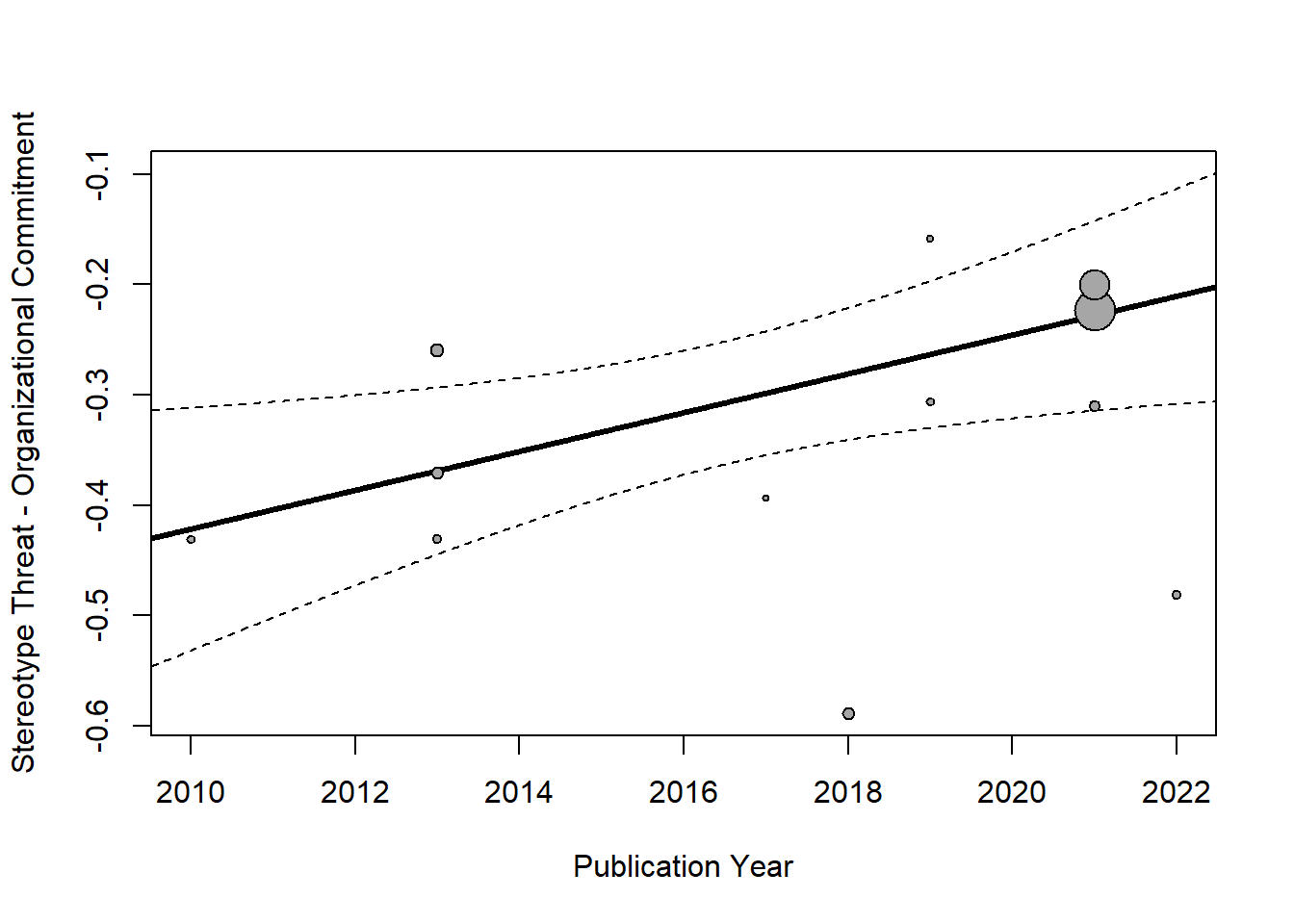** |
| --- | --- |
| **(c) Job Performance**  **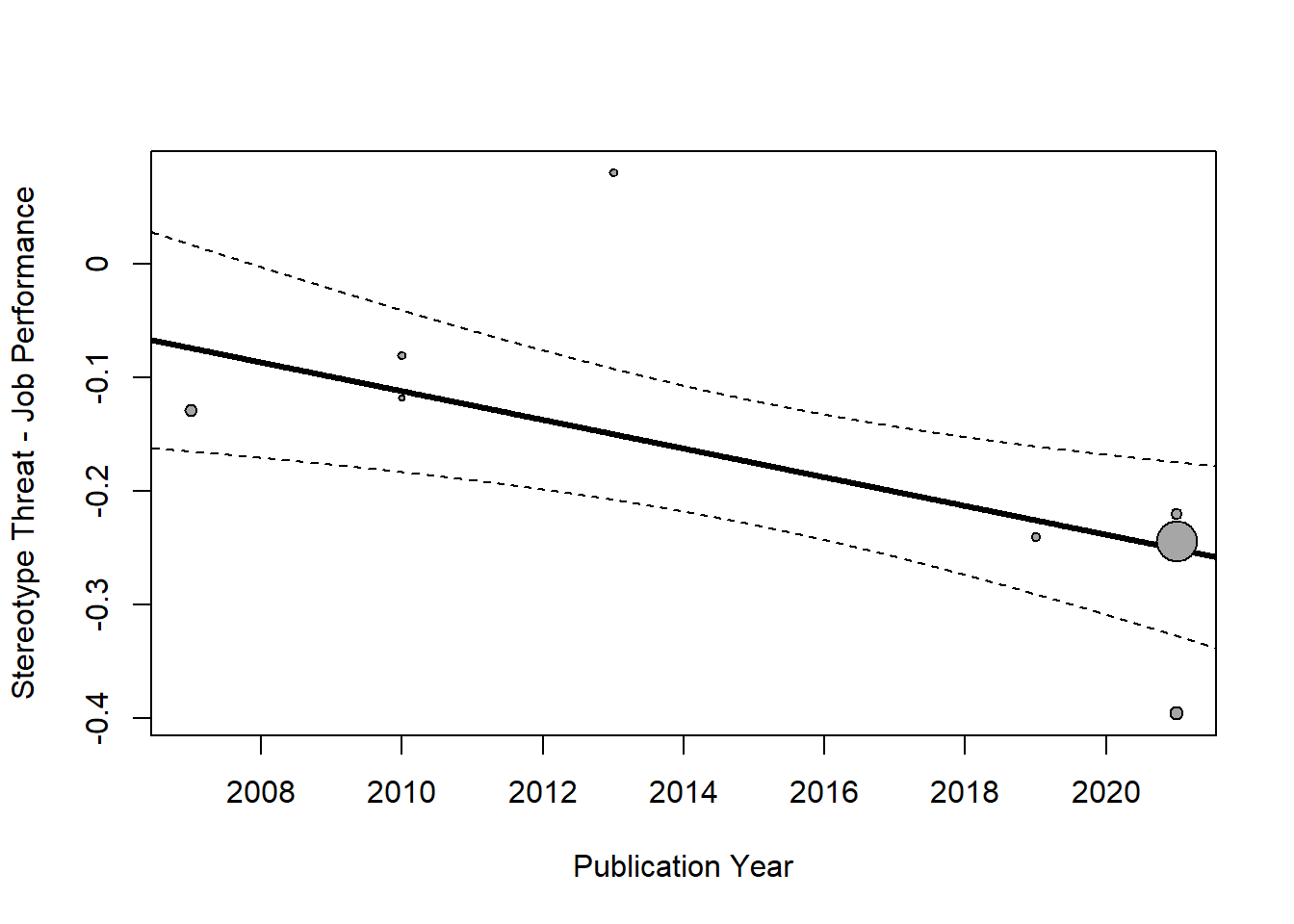** | **(d) Job Satisfaction**  **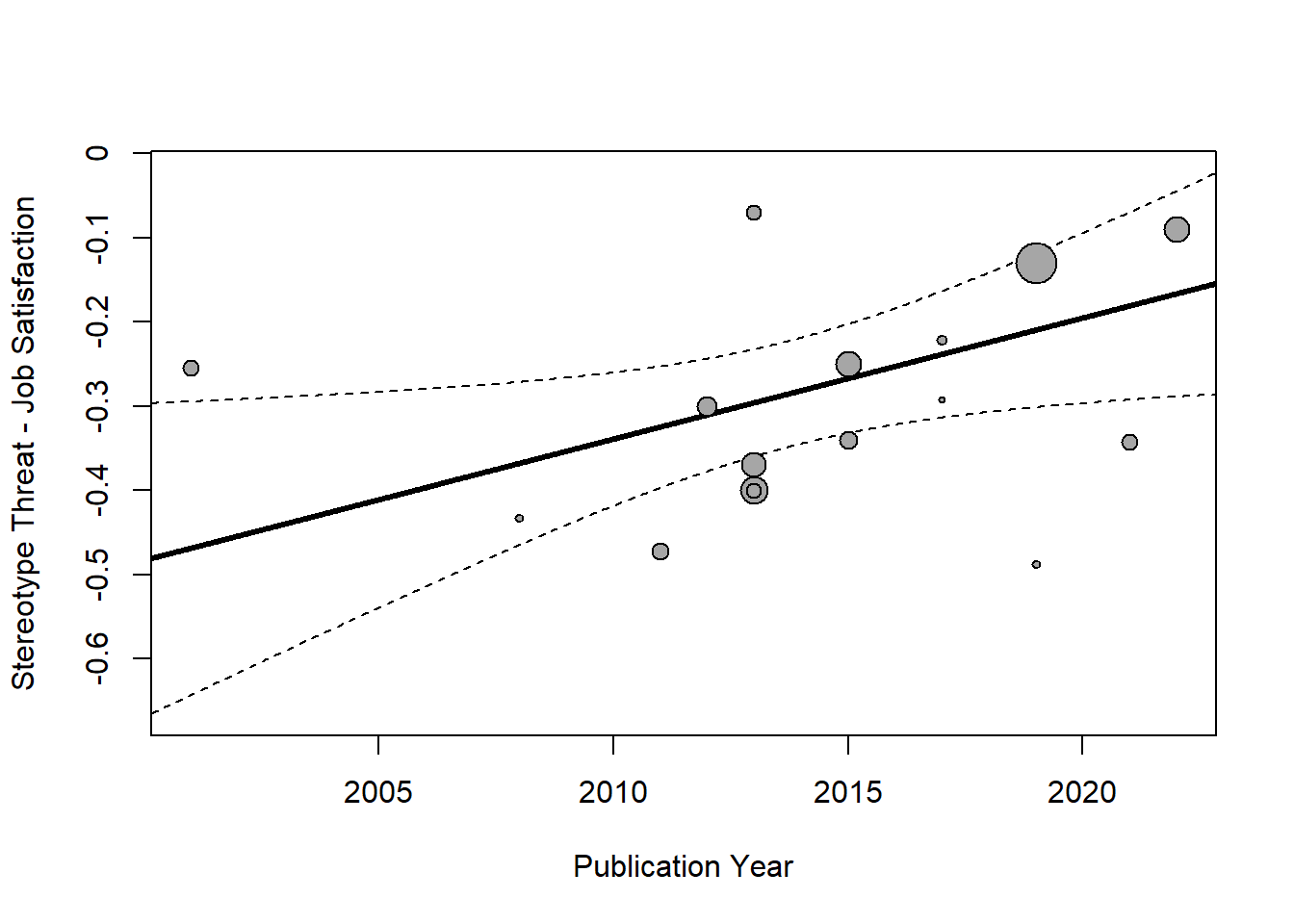** |
